# Fungal immunization potentiates CD4^+^ T cell-independent cDC2 responses for cross-presentation

**DOI:** 10.3389/fimmu.2025.1602174

**Published:** 2025-05-26

**Authors:** Nitish A. Kulkarni, Som G. Nanjappa

**Affiliations:** ^1^ Department of Pathobiology, College of Veterinary Medicine, University of Illinois Urbana-Champaign, Urbana, IL, United States; ^2^ Cancer Center at Illinois, University of Illinois Urbana-Champaign, Urbana, IL, United States

**Keywords:** migratory dendritic cells, conventional dendritic cells, fungal, vaccine, crosspresentation, CD4^+^ T cells

## Abstract

The incidence rates of fungal infections are increasing, especially in immunocompromised individuals without an FDA-approved vaccine. Accumulating evidence suggests that T cells are instrumental in providing fungal immunity. An apt stimulation and responses of dendritic cells are pivotal in inducing T-cell responses and vaccine success. Using a mouse model of fungal vaccination, we explored the dynamics, kinetics, activation, and antigen presentation of dendritic cell subsets to unravel the features of dendritic cell responses in the absence of CD4^+^ T cell help. The subcutaneous fungal vaccination induced more robust cDC2 responses than the cDC1 subset in draining lymph nodes. A single immunization with *Blastomyces* yeasts bolstered DC responses that peaked around day 5 before reverting to basal levels by day 15. The migratory cDC2 was the dominant DC subset, with higher numbers than all other DC subsets combined. Fungal vaccination augmented costimulatory molecules CD80 and CD86 without altering the levels of MHC molecules. Despite the higher fungal antigen uptake with migratory cDC2, the mean cross-presentation ability of all DC subsets was similar. Counterintuitively, deleting CD4^+^ T cells enhanced the DC responses, and CD4^+^ T cells were dispensable for conventional cross-presenting cDC1 responses. Collectively, our study shows that fungal vaccination selectively augmented cDC2 responses, and CD4^+^ T cells were dispensable for DC activation, antigen uptake, expression of costimulatory molecules, and cross-presentation. Our study provides novel insights into DC responses to an effective fungal vaccine for designing efficacious vaccines tailored for immunocompromised hosts.

## Introduction

The global threat of fungal infections is alarming, especially in immunocompromised individuals with dysfunctional or deficient CD4^+^ T cells (1, 2). Fungal infections are deadly and reported to cause case fatality rates ranging 20-90% with more than 3.8 million global deaths annually (3) and significant healthcare burden up to 11.5 billion USD (2). The current antifungal drug arsenal is limited, and its use results in severe organ toxicity, drug resistance, and adverse metabolic effects (4–7). Thus, novel preventive measures, including vaccines to combat fungal infections, are needed to protect vulnerable immunocompromised individuals (8, 9). Despite several lines of preclinical experimental vaccines, there are no FDA-approved fungal vaccines available, but significant strides have been made recently to delineate the mechanisms of effective vaccine immunity.

The development of dendritic cell (DC)-based vaccines has recently gained prominence as a potential avenue to fight against fungal infections (9). DCs are the bona fide innate immune cells that link innate and adaptive immune systems, and they are necessary for inducing robust T-cell responses. Thus, their optimal stimulation is required for vaccine efficacy (10, 11). DCs are involved in antigen uptake, processing, presentation, and priming naïve T cells, thus orchestrating the adaptive immune response (12). Based on the functions and phenotypic markers, DCs are classified into specialized subsets like conventional DCs (cDCs), plasmacytoid DCs (pDCs), and monocyte-derived DCs (moDCs) (13), and lymph node-resident cDC1 and cDC2 are mainly involved in antigen presentation and priming of CD8^+^ and CD4^+^ T cells, respectively (14). The migratory cDC subset, a subclass of cDCs, following the antigen uptake, migrates to draining lymph nodes (dLNs) and presents antigens to CD4^+^ and CD8^+^ T cells or transfers the antigen depot to resident cDC1 or cDC2 (15, 16). Additionally, monocytes can differentiate into monocyte-derived dendritic cells (moDCs) and modulate the ongoing T cell responses (17, 18). Thus, different DC subsets tailor and modulate adaptive immune responses.

CD4^+^ T helper (T_h_) cells play an important role in the priming of cytotoxic CD8^+^ T cells (CTLs) (19). Cognate T_h_ cells interact with antigen-displaying DCs to provide co-stimulatory signals that “license” them to cross-present and activate CD8^+^ T cells effectively (20). Although CD4^+^ T cells can help CD8^+^ T cell responses in many ways (21), the “licensing” of DCs requires their interaction with CD40 on DCs through CD40L (19, 22, 23) and upregulation of costimulatory molecules CD80/86 on DCs to cross-present and activate CD8^+^ T cells (24). Numerous studies in viral and tumor models have shown that cDC1 subsets are well-equipped with cross-presentation ability and activation of CD8^+^ T cells (24–26). Expression of CD40 on cDC1 subsets was essential for their survival, and optimal CD8^+^ T cell responses were governed by CD4^+^ T cell help in allogenic adenovirus transformed cell immunization, tumor inoculation, and HSV-1 and CMV infection models (20, 23, 24, 27). Further, CD4^+^ T cells can indirectly help CD8^+^ T cells by facilitating chemotaxis signals, enhancing inflammation, and secreting IL-2. Thus, CD4^+^ T cells are essential for cross-presentation and enhancement of CD8^+^ T cell responses (27–29). However, CD4^+^ T cell help-independent cross-presentation and CD8^+^ T cell activation are not well understood.

We and others have previously shown that effector CD8^+^ T cells were elicited in the absence of CD4^+^ T cells and compensated CD4^+^ T cells for vaccine-induced immunity to fungal infections (30–37). In the model of blastomycosis during CD4^+^ T-cell deficiency, using an experimental vaccine strain lacking essential virulence factor *Blastomyces* adhesin-1 (B.d. #55) of *Blastomyces dermatitidis* (ATCC #26199) (38), IL-17A-expressing CD8^+^ T cells (Tc17 cells) were necessary, and GM-CSF co-expression further bolstered the vaccine immunity (39, 40). The effector Tc17 cells formed long-lived and stable memory cells without plasticity towards IFNγσυπ+ cells, even in the absence of CD4^+^ T cells (30, 41, 42). Ablation of a negative regulator of T cell signaling, CBLB an E3 Ubiquitin ligase, bolstered the CD8^+^ T cell responses to the heat-killed version of the experimental fungal vaccine on par with the live vaccine strain (43), suggesting the feasibility of enhancing anti-fungal CD8^+^ T cell responses to safer vaccine candidates in the absence of CD4^+^ T cell help. Thus, fungal vaccination induces optimal dendritic cell responses that overcome the CD4^+^ T cell help for activation and programming of CD8^+^ T cell responses. Here, we studied the kinetics, dynamics, activation, and cross-presentation of different dendritic cells to an experimental fungal vaccine. Further, we studied the role of CD4^+^ T cells in regulating the dendritic cell responses, including cross-presentation.

## Materials and methods

### Mice

Wild-type C57BL/6 mice were purchased from Charles River Laboratories (Wilmington, MA) or Jackson Laboratories (Bar Harbor, ME). Mice were housed and used in a specific pathogen-free environment. All experiments were done using both male and female mice of age 6–8 weeks. The animal housing and experiments were performed according to the strict guidelines of the Institutional Animal Care and Use Committee at the University of Illinois Urbana-Champaign.

### Ethics statement

This work was executed in accordance with the protocols approved by the Institutional Biosafety Committee (IBC) and Institutional Animal Care and Use Committee (IACUC) at the University of Illinois at Urbana-Champaign.

### Fungal vaccination

The mice were vaccinated with an isogenic, attenuated (mutant lacking an essential virulence factor, *Blastomyces* adhesion-1; BAD1) (38) strain of *Blastomyces dermatitidis* ATCC 26199 named B.d. #55 (a kind gift from Dr. Bruce Klein, UW-Madison) subcutaneously (s.c.; ~2 × 10^5 CFU) at two sites, dorsally and at the base of the tail. For antigen presentation studies, recombinant B.d. #55 strain engineered to express OT-I epitope SIINFEKL-mCherry (30) was used for vaccination and the cohorts of mice vaccinated with OVA_257–264_ peptide (10 μg/mL) pre-mixed with non-recombinant *Blastomyces dermatitidis* #55 served as positive control. The yeast strains were cultured on Middlebrook 7H10 agar slants supplemented with OADC (oleic acid-albumin complex; Sigma-Aldrich) at 39°C in a humidified incubator.

### Lymph node preparation for DC characterization

The draining lymph nodes (dLN) were harvested and processed as described (44, 45). Briefly, the harvested LN were dissected using 22G needles, and the separated tissues were digested in 1 mg/ml of Collagenase, Type IV (Stem-Cell) in 1X PBS containing 10 μg/ml DNAse I (Roche), kept at 37°C for 30 minutes. Digested tissues/cells were washed with 1X PBS, passed through 40μm strainers (BD Biosciences), and resuspended in complete RPMI media (supplemented with 10% FBS/NEAA/PenStrep). The single-cell suspensions were stained with fluorochrome-conjugated antibodies and analyzed by flow cytometry.

### Labeling of yeast with PKH26 dye

Yeasts were labeled with the Red Fluorescent Cell Linker Dye Kit, PKH26-MIDI26 (Sigma, St. Louis). The yeasts were harvested from the slants and incubated with diluted PKH26 (2 × 10^ (6) yeasts/ml) for 5 minutes at room temperature. To quench excess PKH26 dye, an equal volume of FBS (Corning) was added, mixed, and kept for a minute before washing with complete RPMI medium followed by 1X PBS.

### CD4^+^ T cell depletion

For depletion of CD4^+^ T cells, GK1.5 MAb (BioXCell Inc., Lebanon, NH) was injected intravenously (i.v.) at every 3–4 days with a dose of 200 μg/mouse. The depletion efficiency of CD4^+^ T cells was verified by flow cytometry using MAb Clone RM4-5 (30).

### Flow cytometry

All the antibodies were purchased from BD Biosciences, Biolegend, and Invitrogen. Single-cell suspensions of the dLNs were first incubated with anti-FCγ receptor MAbs (Fc Block; BD Biosciences) for 10 minutes before staining with fluorophore-conjugated antibodies for surface markers and Live/Dead stain (Invitrogen) in FACS buffer (2% BSA in 1X PBS with 0.1% NaN_3_) for 30 minutes on ice. For measuring antigen cross-presentation, single-cell suspensions were stained with anti-mouse H-2K^b^/SIINFEKL antibody (Clone: 25-D1.16) during surface staining for markers. The stained cells were washed three times with FACS buffer and fixed with 2% Paraformaldehyde in 1X PBS. The cells were analyzed using a full-spectrum flow cytometer, Cytek Aurora. The data were analyzed with FlowJo v10.10 (BD Biosciences).

### Statistical analysis

Statistical analyses of DC dynamics, activation status, and antigen presentation of DC subsets in the unvaccinated vs. vaccinated mice and the presence versus absence of CD4^+^ T cells were conducted using a two-tailed unpaired Student’s t-test. DC kinetics and fungal antigen uptakes were analyzed by one-way ANOVA with *post-hoc* tests of Dunnett’s correction and Tukey’s correction for multiple comparisons, respectively. We performed all the statistical analyses using Prism 10.2 (GraphPad Software, LLC) software. A two-tailed p-value of ≤ 0.05 was considered statistically significant.

## Results

### Fungal vaccination preferentially expanded the type 2 dendritic cells

**Figure 1:**
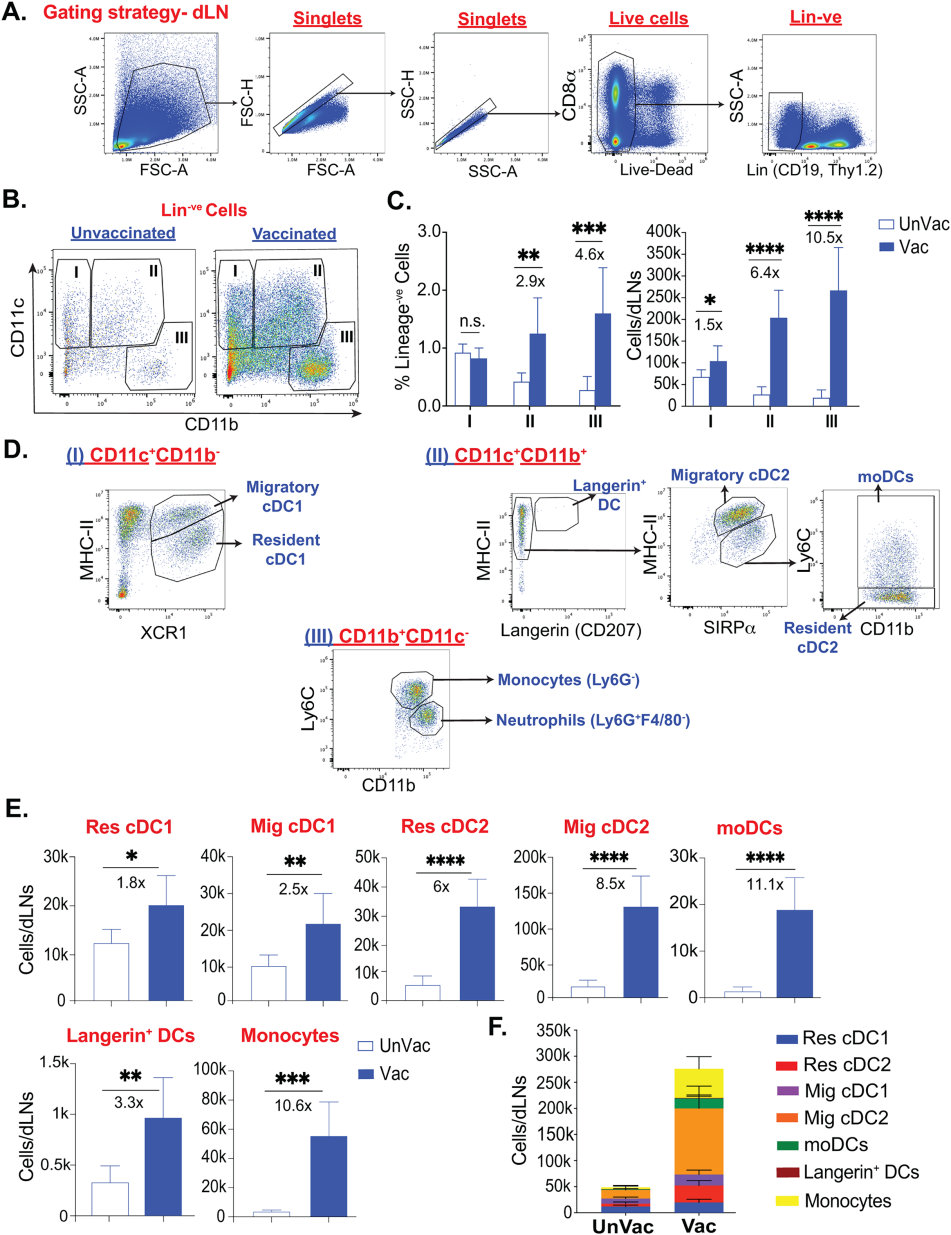
Fungal vaccination significantly enhanced the populations of dendritic cell subsets in draining lymph nodes following subcutaneous fungal vaccination. Naïve C57BL/6 mice, upon CD4^+^ T-cell depletion, were vaccinated subcutaneously with live attenuated strain (#55) of *Blastomyces dermatitidis* (~2x10^5^) CFUs). On day 5 post-vaccination (D5PV), draining lymph nodes were harvested to obtain a single-cell suspension. Following antibody staining, the cells were analyzed by flow cytometry. **(A)** Gating strategy to obtain Live^+^Lineage^-ve^ (non-Thy1.2, CD19) cells. **(B)** Flow plots show populations I (CD11c^+^CD11b^-^), II (CD11c^+^CD11b^+^), and III (CD11c^-^CD11b^+^) among Lineage^-ve^ cells in unvaccinated and vaccinated C57BL/6 mice. **(C)** Frequencies among Lineage^-ve^ cells and absolute cell numbers of populations I, II, and III. **(D)** Further gating includes population I, which consists of resident cDC1 and migratory cDC1. Population II contains langerin^+^ DCs, resident cDC2, migratory cDC2, and Ly6C^+^moDCs, while monocytes and neutrophils are gated among population III. **(E)** Absolute cell numbers of resident cDC1, migratory cDC1, resident cDC2, migratory cDC2, moDCs, Langerin^+^ DCs, and monocytes in unvaccinated and vaccinated CD4^-^ mice at D5PV. **(F)** Stacked column graph depicts absolute numbers of overall DC subsets in unvaccinated and vaccinated mice at D5PV. Data are representative of at least four independent experiments. N=5-8 mice/group. Values are in Mean ± SD. p*≤0.05, p**≤0.01, p***≤0.001, and p****≤0.0001, unvaccinated versus vaccinated WT. Mice were injected with GK1.5 (200 μg/mouse) throughout the experiment to deplete CD4^+^ T cells. DC, dendritic cell; cDC, conventional dendritic cell; moDC, monocyte-derived dendritic cell; Res, resident; Mig, migratory; Unvac, unvaccinated; Vac, vaccinated.

The immunization routes and the nature of the antigen or pathogen dictate the dominant type of T cell responses associated with the changes in the dendritic cell subsets (11). For example, in a viral infection, where most DCs could be infected, the T cell priming can occur at many regions of the lymph node, and cDC1-lineage predominantly shapes CD8^+^ T cell responses (46, 47), possibly due to their preferential niche or migration into the deep T cell zone (48). Further, several studies have shown that cDC1-lineage dendritic cells are required for cross-presentation (25). Previously, we have shown that antifungal effector and memory CD8^+^ T cells can be induced by subcutaneous route immunization using an experimental vaccine in the absence of CD4^+^ T cells and mediate sterilizing immunity to lethal pulmonary infection (30, 39–41). We noted that the vaccine immunity (efferent phase) directly correlated with the nature and magnitude of effector CD8^+^ T cells generated in the skin-draining lymph nodes (*afferent phase*) *(* 39, 41, 49). However, the dendritic cell subset responses that may dictate antifungal effector CD8^+^ T cell-responses during *afferent phase* are not known. Therefore, we wanted to determine the dynamics of dendritic cell populations that may dictate the cross-presentation following fungal vaccination during CD4^+^ T cell deficiency during afferent phase. At day 5 post-vaccination, we harvested the draining lymph nodes (dLN), and single-cell suspensions were stained and analyzed by flow cytometry. We classified dendritic cell populations broadly into two groups (I and II) based on the expression levels of CD11c and CD11b (16, 50) (**Figures 1A, B**). When we compared with naïve, non-immunized, controls, the fungal vaccination significantly enhanced the numbers of Groups I and II cells but not the frequency of Group I cells (**Figure 1C**). We found Group I cells mainly composed of migratory cDC1 and resident cDC1 cells and Group II cells comprised primarily of Langerin^+^ DC, migratory cDC2, resident cDC2, and monocyte-derived DCs (moDCs) (13, 51, 52) (**Figure 1D**). CD11c^-^CD11b^+^ cells (Group III), mainly consisted of monocytes and neutrophils, significantly increased following vaccination (**Figures 1C, D**). We used XCR1 and SIRPα (CD172a) markers to classify type-1 and -2 cDCs, respectively (51). Additionally, we used the expression of CD11c and MHC-II to classify DC into resident cDCs (CD11c^high^MHC-II^int^) and migratory cDCs (CD11c^int^MHC-II^high^) (52, 53). The moDCs and pDCs subsets were classified as CD11c^+^Ly6C^+^CD11b^+^MHC-II^int/lo^ and CD11c^lo^CD11b^-^Ly6C^+^MHC-II^lo^, respectively (13, 54, 55). Since XCR1 and SIRPα can reliably be used to classify cDC1 and cDC2, and XCR1^+^ DCs are instrumental in cross-presentation (56–58), we further evaluated the dynamics of different DC subsets following vaccination. We found an increase in the numbers and frequencies of most subsets of resident and migratory cDC1 and cDC2 cells in vaccinated mice compared with an unvaccinated group (**Figure 1E**; **Supplementary Figure 1**). Notably, the numbers of cDC2 cell subsets were significantly increased compared with a modest increase of cDC1 cells (~6-8X vs. ~2X) following vaccination. The order of significant increase of dendritic cell types following the vaccination was migratory cDC2, resident cDC2, migratory cDC1, and moDCs, followed by resident cDC1 and Langerin^+^ DC (**Figure 1F**). Collectively, the fungal vaccination significantly augmented cDC2 over cDC1-type dendritic cells.

### cDC2, but not cDC1, subsets were enriched during the early phase of fungal vaccine response

**Figure 2:**
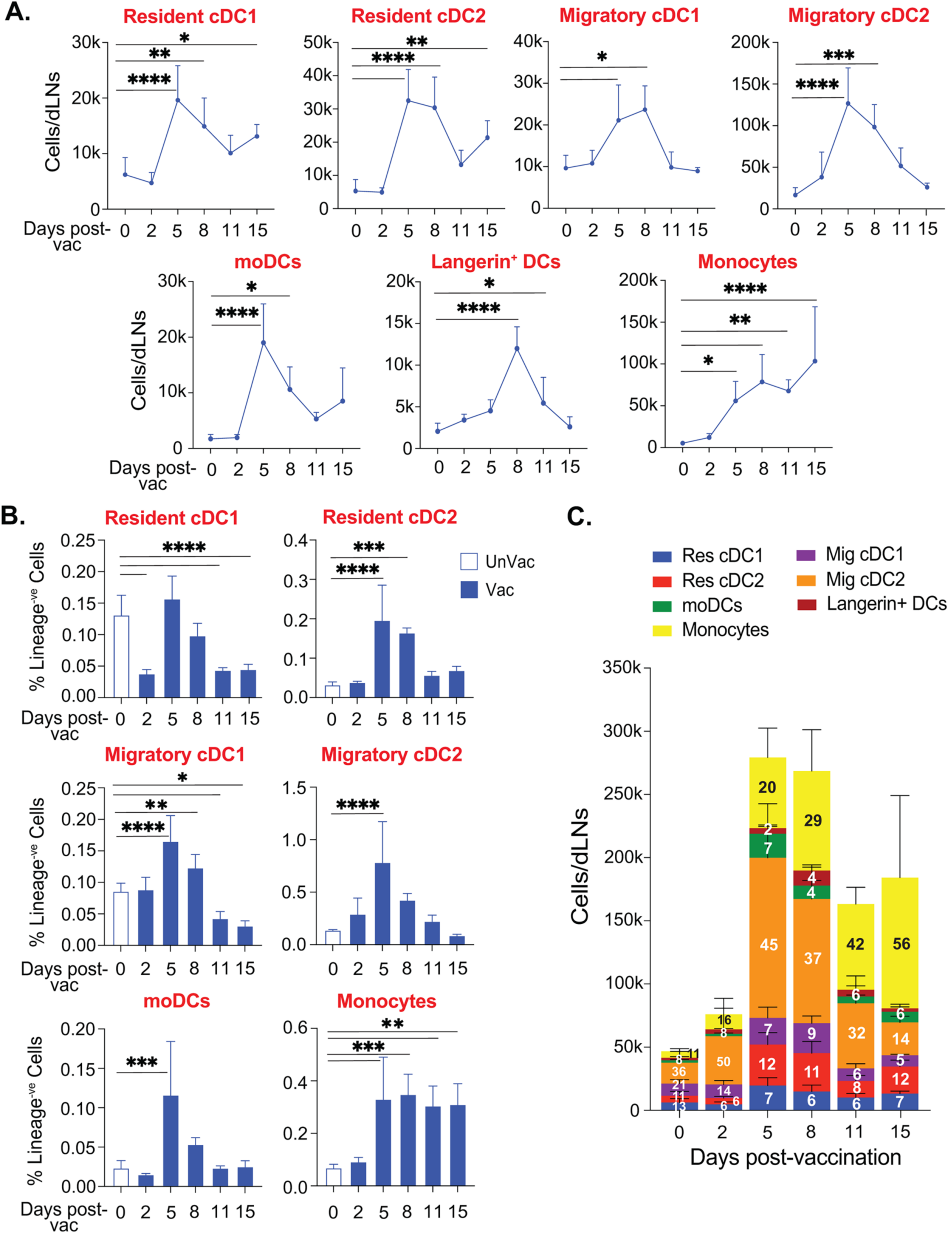
cDC2 expanded during the early phase of subcutaneous fungal vaccination. Naïve CD4^-^depleted C57BL/6 mice were vaccinated subcutaneously with live attenuated strain (#55) of *Blastomyces dermatitidis* (~2×10^5 CFUs). On days 0, 2, 5, 8, 11, and 15 post-vaccination, dLNs were harvested to analyze the kinetics of various DC subsets by flow cytometry. **(A)** The absolute cell number kinetics and **(B)** frequency (among lineage^-ve^ cells) kinetics of resident cDCs, migratory cDCs, monocyte-derived DCs, and monocytes in unvaccinated and vaccinated mice. **(C)** Stacked column graph depicts absolute cell numbers and proportions (within time-point) of overall DC subsets in unvaccinated and vaccinated mice at different time points post-fungal vaccination. Data are representative of at least two independent experiments for the indicated days post-vaccination. p*≤0.05, p**≤0.01, p***≤0.001, and p****≤0.0001, comparison of different time points with unvaccinated (day 0) control. N=5-8 mice/group/time-point. Values are in Mean ± SD. Mice were injected with GK1.5 (200 μg/mouse) throughout the experiment to deplete CD4^+^ T cells. dLN, draining lymph node.

The subcutaneous route of immunization with large microbial antigens will induce migratory cells to engulf antigens and shuttle them to draining lymph nodes, an orchestrated process distinct from the quick passive transfer of soluble small antigens (59). Skin or local DCs play a larger role in shuttling the antigen cargo into the draining lymph nodes for antigen deposition and presentation. Since several dendritic cells carry the antigen cargo from the subcutaneous space and influence the resident and hematopoietic cell-derived dendritic cell populations for antigen presentation and T cell activation in a time-dependent manner, we determined the kinetics of dendritic cell subsets following subcutaneous vaccination in the draining lymph nodes. As expected, we did not find major changes in the resident cDC subsets at day 2 post-vaccination (**Figures 2A, B**). However, there was a significant increase in their numbers by day 5 post-vaccination (PV) that stabilized till day 8 before tapering off. The number of resident cDC subsets remained elevated even at day 15 PV. In contrast, migratory DCs and Langerin^+^ DC numbers were steadily increased starting as early as day 2 PV, peaking around days 5 to 8 PV before reducing to the basal levels by day 15 PV (**Figures 2A, B**). The kinetics of moDCs were similarly reflected. Not surprisingly, the kinetics of the monocyte population were distinct and steadily increasing through day 15 PV. Although the overall kinetics of various subsets of the dendritic cell population mirrored each other in the dLN (**Figure 2B**; **Supplementary Figure 2**), the numbers of each subset were noticeably different. Therefore, we evaluated the relative abundance of dendritic cell subsets, including monocytes, over time, which may reflect their ability to engage with the T cells. The relative proportions of potent cross-presenting DC subsets and resident/migratory cDC1 subsets were stable or decreased following the vaccination (**Figure 2C**). The significant and most abundant DC cell subset was migratory cDC2 that started to inflate as early as day 2 PV and remained as such till day 8 PV and was replaced by accumulating CD11b^+^Ly6C^+^ monocytes. By day 15 PV, CD11b^+^Ly6C^+^ monocytes were the most abundant cells. Interestingly, resident cDC2, rather than conventional cross-presenting cDC1, population was the third most abundant cell type throughout the vaccine response period.

Collectively, cDC2, but not cDC1, subsets were abundant in the early phase of vaccine response and gradually replaced by the monocytic population later.

### Fungal vaccination differentially induces the activation of resident cDC, migratory cDC, and moDC

**Figure 3:**
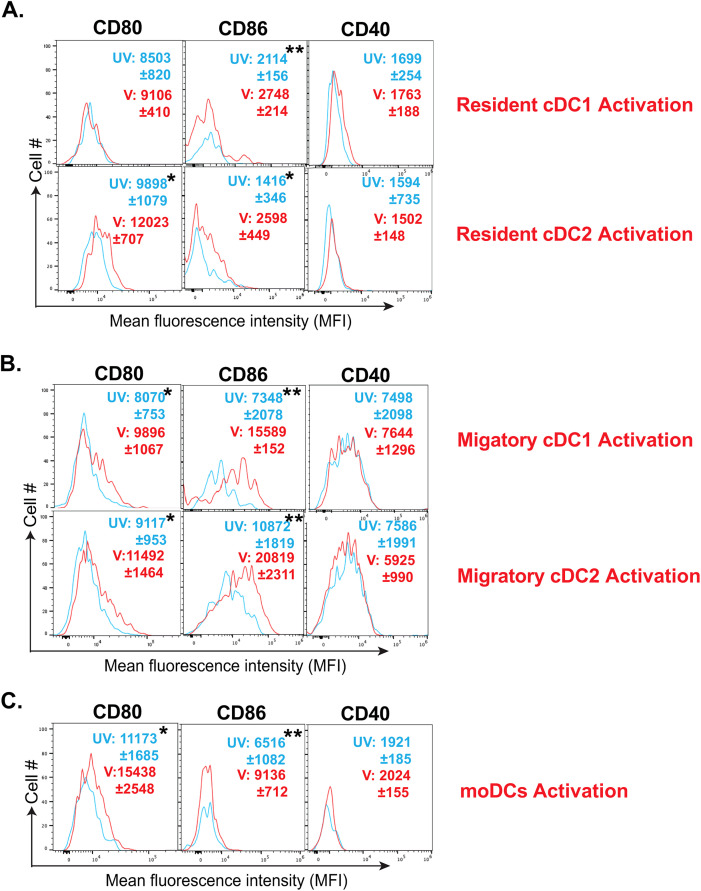
Fungal vaccination increased the activation of resident cDCs, migratory cDCs, and moDCs. The activation status of various DC subsets in dLNs of CD4^-^ C57BL/6 mice following fungal vaccination was determined by flow cytometry. The activation status of **(A)** resident cDC1, resident cDC2, **(B)** migratory cDC1, migratory cDC2, and **(C)** monocyte-derived DCs was marked by the expression (MFI) of co-stimulatory molecules such as CD80, CD86, and CD40 at day 5 post-vaccination, depicted in overlay histograms. The cyan histogram shows unvaccinated mice, and the red histogram depicts vaccinated mice. Data are representative of at least two independent experiments. N=3-5 mice/group. MFI values are in Mean ± SD. p*≤0.05, and p**≤0.01. Mice were injected with GK1.5 (200 μg/mouse) throughout the experiment to deplete CD4^+^ T cells. dLN, draining lymph node; UV, unvaccinated; V, vaccinated; MFI, mean fluorescent intensity.

DC status largely dictates its phagocytosis, antigen presentation, and the activation of T cells’ abilities. While immature DCs are highly phagocytic, their activation and maturation are accompanied by higher expression of costimulatory and MHC molecules optimized for activation of T cells (60). Here, we evaluated the expression of different costimulatory molecules and MHC molecules on different dendritic cell subsets following fungal vaccination. We found that vaccination induced the upregulation, albeit in a disparate manner, of CD80 and CD86 molecules on resident DC subsets (**Figure 3A**). Similarly, the upregulation of costimulatory molecules, CD80 and CD86, was significant on migratory DC and moDC subsets following vaccination (**Figures 3B, C**) However, the expression of the costimulatory molecule CD40 remained unaltered following the vaccination in all the subsets. Despite DCs constitutively expressing high levels of MHC-I and MHC-II molecules (61), their expression may increase following their activation (62). However, we did not find significant upregulation of either of the MHC molecules on any DC subsets (**Figures 3A, B**). Thus, our data suggest that fungal vaccination efficiently fosters the activation of DC subsets by upregulating CD80 and CD86 but not CD40 molecules in the absence of CD4^+^ T cells.

### Different dendritic cell subsets efficiently uptake the fungal antigens and present them following vaccination

**Figure 4:**
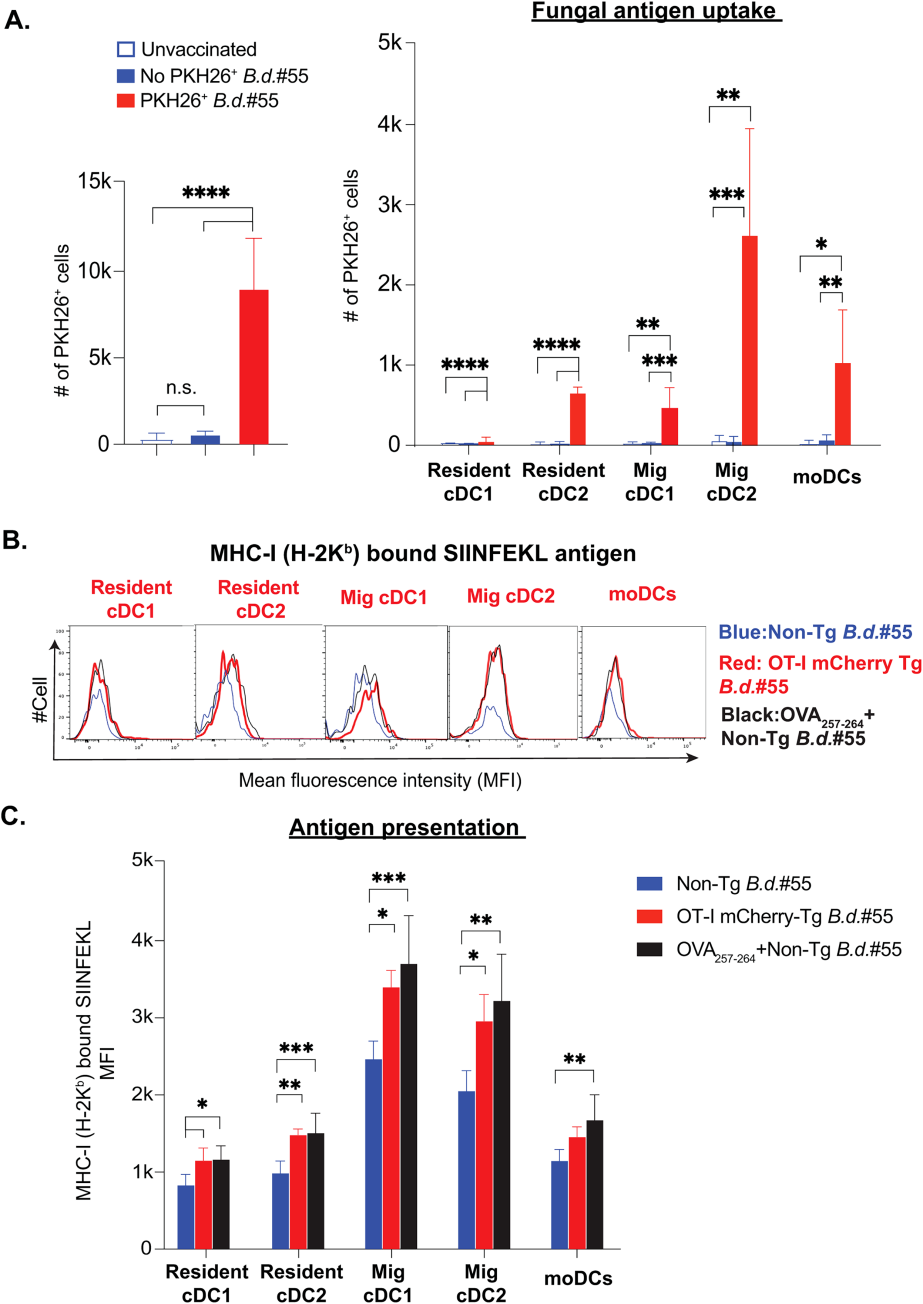
Dendritic cell subsets effectively uptake fungal antigens and cross-present following fungal vaccination. **(A)** Naïve CD4^-^ were subcutaneously vaccinated with PKH26^+^ labeled or unlabeled live attenuated strain (#55) of *Blastomyces dermatitidis* (~6-8x10^6^) CFUs). The bar graphs show the overall number of PKH26^+^ yeast cells among Live^+^Lineage^-ve^ dLN cells at day 5 post-vaccination. The grouped bar graphs depict the distribution of PKH26^+^ yeast among the DC subsets in dLNs, shown in absolute numbers at day 5 post-vaccination. Values denote the mean ± SD of at least two independent experiments from N=3-5 mice/group. p*≤0.05, p**≤0.01, p***≤0.001, and p****≤0.0001, analyzed by one-way ANOVA with Tukey’s multiple comparison test. **(B, C)** The cohorts of naïve CD4 depleted C57BL/6 mice were vaccinated subcutaneously with non-transgenic (live attenuated strain #55) *Blastomyces dermatitidis* (Blue) or mCherry (OT-I #55) transgenic *Blastomyces dermatitidis* (Red) or OVA_257-264_ incubated non-transgenic yeast (Black) (~2x10^5^ CFUs). **(B)** The overlay histograms and **(C)** grouped bar diagrams depict the cross-presenting ability of the SIINFEKL antigen by DC subsets. This is quantified by flow cytometry staining of dLNs with 25-D1.16 monoclonal antibody that recognizes the SIINFEKL-H-2K^b^ complex at day 5 post-vaccination. Data are representative of at least two independent experiments. N=4-6 mice/group. Number and MFI values are in Mean ± SD. p*≤0.05, p**≤0.01, p***≤0.001, and p****≤0.0001, analyzed by one-way ANOVA with Tukey’s multiple comparison test. dLN, draining lymph node. Mice were injected with GK1.5 (200 μg/mouse) throughout the experiment to deplete CD4^+^ T cells. MFI, mean fluorescent intensity.

Our aforementioned data suggested that fungal vaccination predominantly enhances the cDC2 subsets and expression of CD80 and CD86 costimulatory molecules on most DC subsets. Antigen uptake is an essential event before presentation in the context of MHC molecules, and immature DCs readily engulf large antigens by phagocytosis. Subcutaneously inoculated antigens are picked up by local dendritic cells, which facilitates their migration to the draining lymph nodes as part of migratory DC subsets. Although several overlapping markers are noted between migratory and lymph node resident DC subsets, making it difficult to distinguish, migratory DCs can transfer the antigen cargo to tissue-resident DC subsets. Our data suggested that PKH-labelled fungal antigen is readily detected by flow cytometry, which enabled us to assess fungal antigen-engulfed DC subsets (**Figure 4A** & **Supplementary Figure 4A**). The fungal antigens were found in all the subsets, but predominantly in migratory cDC2 subset, followed by migratory cDC1 and moDCs (Supplementary **Figure 4B**), in line with their augmented numbers at day 5 post-vaccination (**Figure 2C**). Next, we evaluated their ability to cross-present the fungal antigens. The MAb staining for SIINFEKL-bearing MHC-I (H-2K^b^) following vaccination with non-Tg and Tg yeasts showed the utility of the MAb to assess the presentation (**Figure 4B**). Using this technique, we assessed the cross-presenting ability of different DC subsets (**Figure 4C**) and found that all DC subsets were able to cross-present the fungal antigens, with a modestly higher level by migratory cDCs and lower by moDCs.

Collectively, despite all DC subsets being able to cross-present, the migratory cDC2 population predominates in gorging and presenting the fungal antigens.

### CD4^+^ T cell depletion augments the activation and numbers of cDC2 and moDC subsets

**Figure 5:**
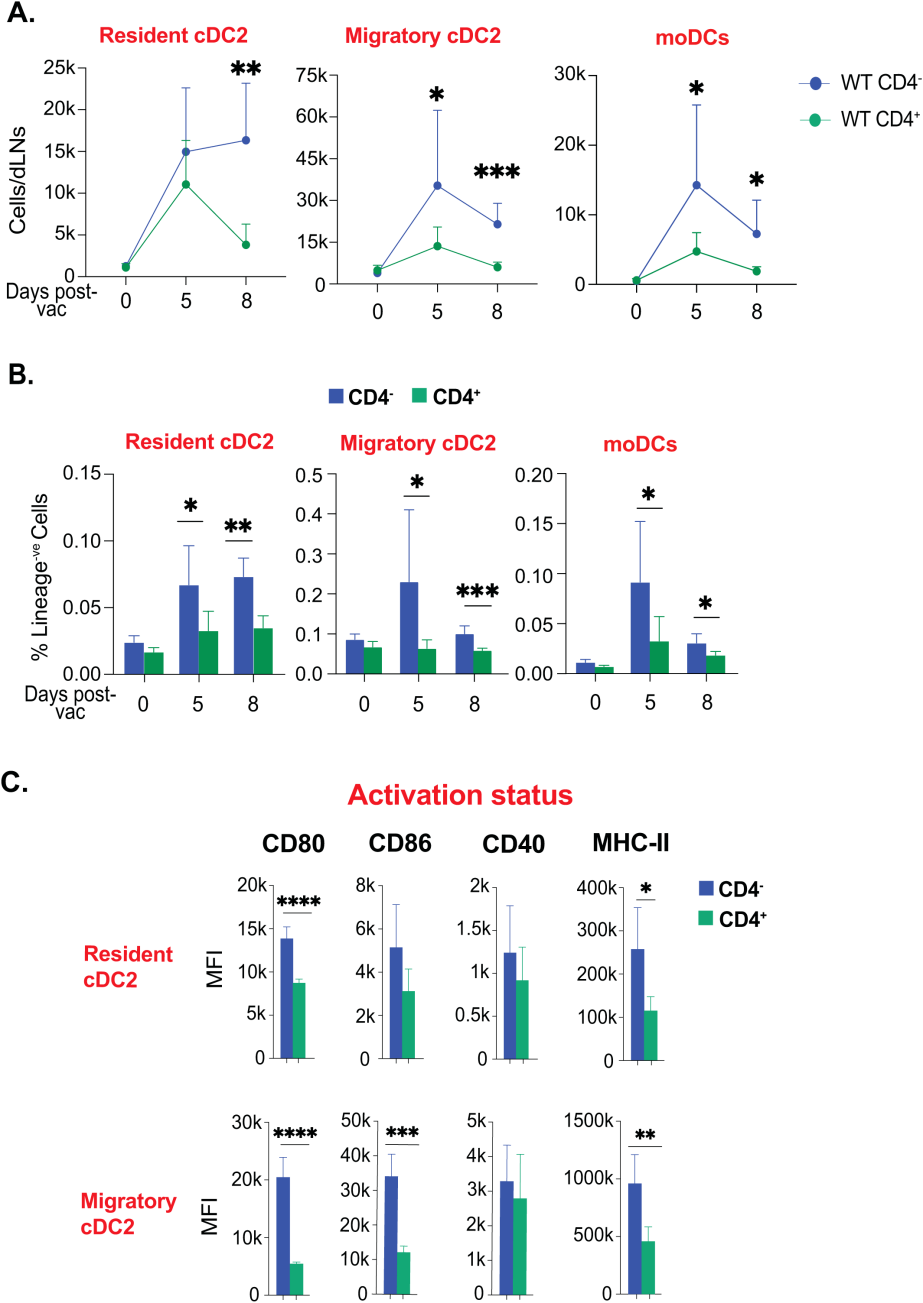
Ablation of CD4^+^ T-cells enhanced the kinetics and activation status of cDC2 and moDCs. Naïve CD4^+^ and CD4 depleted C57BL/6 mice were vaccinated subcutaneously with live attenuated strain (#55) of *Blastomyces dermatitidis* (~2x10^5^ CFUs). On indicated days post-vaccination, dLNs were harvested to analyze various DC subsets by flow cytometry. The kinetics of **(A)** cell number and **(B)** frequencies (among lineage^-ve^ cells) of resident cDC2, migratory cDC2, and moDCs are depicted. Values are Mean ± SD. **(C)** The bar graphs depict the activation status of resident cDC2, migratory cDC2, and moDCs marked by the expression of co-stimulatory molecules (CD80, CD86, and CD40) and surface marker MHC-II (I-A/I-E) at day 5 post-vaccination. N=3-5/mice/group/time-point. Data are representative of at least two independent experiments. Percent, numbers, and MFI values are in Mean± SD. p*≤0.05, p**≤0.01, p***≤0.001, and p****≤0.0001. Groups of mice were injected with GK1.5 (200 μg/mice) throughout the experiment to deplete CD4^+^ T cells. dLN, draining lymph node; moDC, monocyte-derived dendritic cell.

Our data so far suggested that cDC2 and moDC subsets were predominantly enriched with higher activation in the model of fungal vaccination. We asked if cDC2 and moDC subsets are enriched due to CD4 depletion. We evaluated the kinetics of cDC2 and moDC subsets. We found that despite there being an increase in these subsets in the CD4^+^ T cell-sufficient group, the CD4^+^ T cell-deficient group had augmented numbers that were sustained through days 5 to 8 post-vaccination (**Figure 5A**). Similarly, the frequency and numbers of monocytes were significantly increased in the CD4^+^ T cell-deficient group compared with the sufficient group (**Supplementary Figures 5A, B**). Similarly, the frequency of cDC2 subsets and moDCs was significantly lower in the CD4^+^ T cell-sufficient group than in the deficient group (**Figure 5B**). Since CD4^+^ T cells are known to help activation of dendritic cells, we evaluated the upregulation of costimulatory molecules on dendritic cells. We found that the presence of CD4 T cells did not help in upregulating costimulatory molecules, including CD40 and MHC-II (**Figure 5C**; **Supplementary Figure 5C**). Since the expression of MHC-I is essential for augmenting CD8^+^ T cell responses, we measured its expression levels. We found similarities in dendritic cell subsets of both CD4^+^ T cell-sufficient and -deficient groups (**Supplementary Figure 5D**). To exclude the possibility of depletion-independent antibody effects of GK1.5 MAb, we used isotype control MAb, and we found non-significant effects of depletion-independent functions of MAb on dendritic cell responses to the immunization.

Thus, the depletion of CD4^+^ T cells did not alter the kinetics but the dynamics and activation of cDC2 and moDC subsets.

### CD4^+^ T cell help is dispensable for conventional cross-presenting cDC1 responses following fungal vaccination

**Figure 6:**
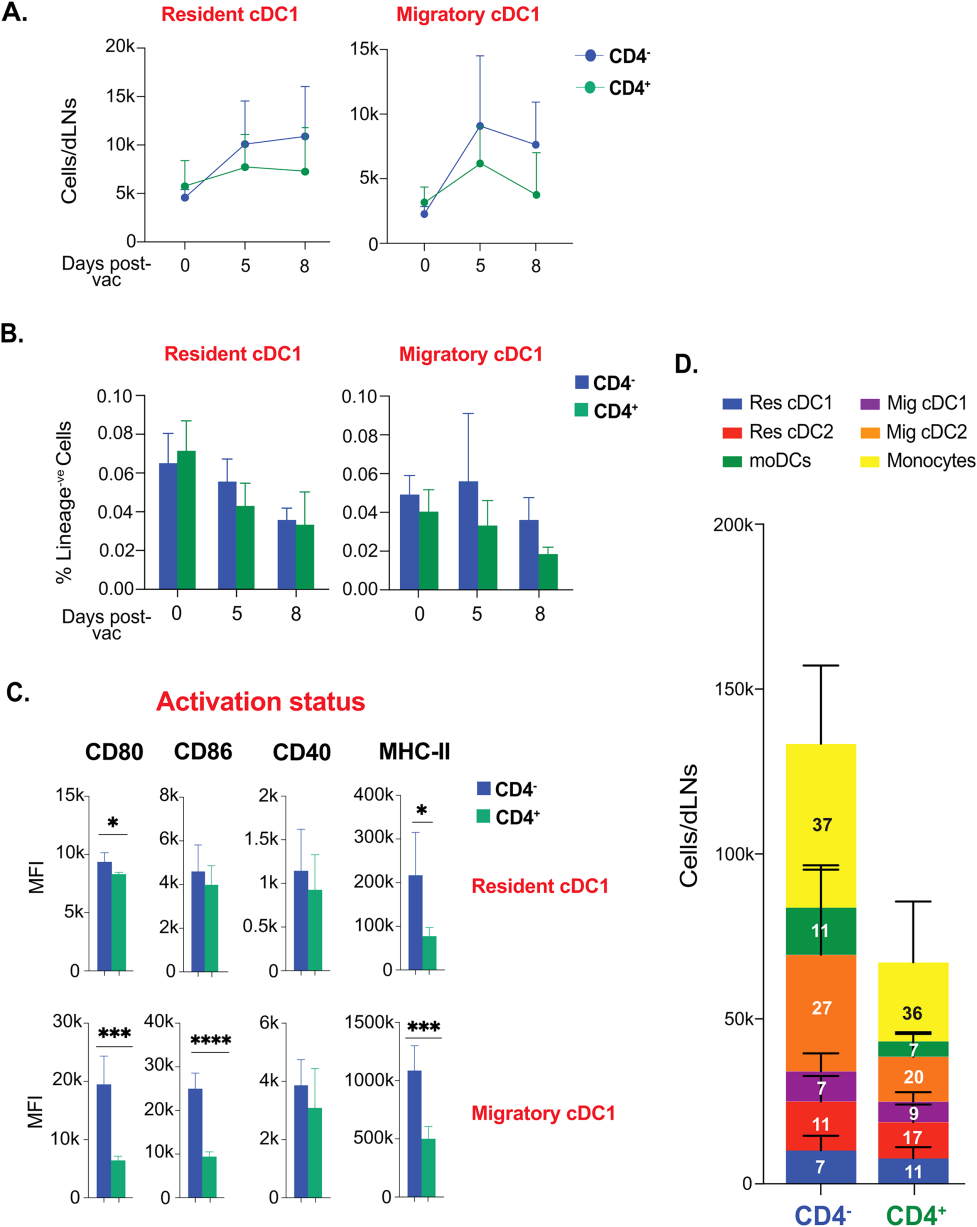
CD4^+^ T cell help is not required for the activation and kinetics of conventional cross-presenting cDC1 subsets following fungal vaccination. Naïve CD4^+^ and CD4 depleted C57BL/6 mice were vaccinated subcutaneously with live attenuated strain (#55) of *Blastomyces dermatitidis* (~2x10^5^ CFUs). On indicated days post-vaccination, dLNs were harvested to analyze various DC subsets by flow cytometry. The kinetics of **(A)** cell number and **(B)** frequencies (among lineage^-ve^ cells) of resident cDC1 and migratory cDC1 are depicted. Values are Mean ± SD. **(C)** The bar graphs depict the activation status of resident cDC1 and migratory cDC1 marked by the expression of co-stimulatory molecules (CD80, CD86, and CD40) and surface marker MHC-II (I-A/I-E) at day 5 post-vaccination. **(D)** Stacked column graph depicts absolute numbers and proportions of DC subsets in CD4^+^ and CD4^-^ vaccinated WT at day 5 post-fungal vaccination. N=3-5/mice/group/time-point. Data are from at least two independent experiments. Percent, numbers, and MFI values are in Mean± SD. p*≤0.05, p**≤0.01, p***≤0.001, and p****≤0.0001. Groups of mice were injected with GK1.5 (200 μg/mice) throughout the experiment to deplete CD4^+^ T cells. moDC, monocyte-derived dendritic cell; Res, resident; Mig, migratory; Unvac, unvaccinated; Vac, vaccinated.

Since CD4^+^ T cells “license” cDC1 for cross-presentation of exogenous antigen, we next evaluated if CD4^+^ T cells are required to bolster the cDC1 that are relevant to the model of fungal vaccination targeted to elicit CD8^+^ T cell responses. The kinetics of the resident and migratory cDC1 subsets remained similar in CD4^+^ T cell-sufficient and -deficient groups through day 8 post-vaccination (**Figure 6A**). Further, the frequency of cDC1 subsets were relatively similar in CD4^+^ T cell-sufficient and -deficient groups at days 0, 5, and 8 post-vaccination (**Figure 6B**). Notably, the expression of classical licensing costimulatory molecule, CD40, on cDC1 subsets, were similar in CD4^+^ vs. CD4^-^ groups (**Figure 6C**). However, the activation status of cDC1 subsets were higher in CD4^+^ T cell-deficient group than in CD4^+^ T cell-sufficient group. We further comprehensively analyzed the landscape of different DC subsets in the CD4^+^ T cell-deficient group, contrasting with the CD4^+^ T cell-sufficient group (**Figure 6D**). We found that proportions of the DC subsets remained largely similar, with a significant increase in cDC2 subsets in the CD4^+^ T cell-deficient group.

Collectively, CD4^+^ T cell help is dispensable following fungal vaccination to augment dendritic cell responses.

## Discussion

Dendritic cells are essential to bridge innate immunity with adaptive immune responses, and understanding dendritic cell responses to fungal vaccines helps design and develop efficacious vaccines. This study addressed four key questions regarding fungal vaccine eliciting the dendritic cell responses: What is the predominant vaccine-elicited DC subset? Do DC subsets have dissimilar cross-presentation abilities to an experimental fungal vaccine? Whether CD4^+^ T cell help is required for cDC1 responses for cross-presentation? Does an experimental fungal vaccine overcome the need for CD4^+^ T cell help? Our study showed that fungal vaccination without CD4^+^ T cells disparately enhances different DC subsets, driving pronounced cDC2 responses. Fungal vaccination induced the activation and upregulation of classical costimulatory molecules CD80 and CD86, but not CD40 and MHC molecules, and CD4^+^ T cells were dispensable for dendritic cell responses and antigen presentation.

Dendritic cell responses to different antigens/immunogens vary and are largely dictated by the nature of the antigen, adjuvant, and soluble vs. cell-associated. Despite the plasticity and the involvement of different subsets of DCs, intracellular pathogens largely drive cDC1 responses, whereas cDC2 responses dominate during allergen exposure, parasites, and other extracellular pathogens. Pattern recognition receptors (PRRs) signaling upon recognition with PAMPs may conceivably intensify and diversify cDC1 vs. cDC2 responses. For example, combining an antigen with poly (I:C) or type B CpG ODN potentiates cDC1 responses (63, 64), helping robust Th1 and CD8^+^ T cell responses. Similarly, many viral infections boost cDC1 responses (65, 66). However, aeroallergen sensitization primarily drove cDC2 response and allergic responses (67). Although all the DC subsets, including moDCs, were significantly enhanced following vaccination in our study, we found a dominant cDC2 response, only replaced by monocytes during the second week following immunization. Although infection with *Aspergillus fumigatus* induces predominant type 1 T cell responses, it induced cDC2 responses in the model of allergy sensitization (68). In line with this, the model of dermal candidiasis using Batf3^-/-^ mice showed cDC1 dispensability for immunity (69), whereas cDC1 was important during pulmonary histoplasmosis and systemic candidiasis (70, 71). Nevertheless, our model system was deficient in CD4^+^ T cells, and CD8^+^ T cell responses are cross-presentation dependent (whole cell-based antigen). Thus, future studies may help understand the relative contributions of cDC1 and cDC2 subsets for inducing CD8^+^ T cell responses.

The efficient cell-associated antigen uptake and transport of antigen cargo by DCs to draining lymph nodes are necessary for eliciting T cell responses (9, 11). Our study showed that migratory cDC2 was the predominant subset in the uptake of fungal antigens. ~70-80% of migratory cDC2 were PKH26^+ve,^ followed by migratory cDC1 and moDCs, and were correlated with their frequency and numbers. Similarly, several others have shown that migratory cDC and moDC mainly uptake tumors, eukaryotic pathogens, and viral antigens (16, 48, 50, 72). Although our model requires cross-presentation for efficient CD8^+^ T cell responses (39, 40), we did not see cDC1 as a predominant subset, including antigen uptake. Nevertheless, mig cDC1 and moDCs were significantly increased following vaccination, but the question remains if they are dispensable due to a compensatory role by other DCs. The resident cDC1 showed lower efficacy of antigen uptake, which may be due to the predominant positioning of resident cDC1 in the deeper medullary region of dLN (11, 73). The efficient cDC2 role in antigen uptake may be due to the nature of antigens and their localization in the cortex of the LN near the afferent lymphatics (11, 73). Further, subcutaneous immunization may predominate with antigen-engulfed cDC2 due to the inherent scarcity of dermal cDC1; thus, fewer cDC1 cells are likely to engage in the uptake of antigens (74, 75). However, soluble antigens can equally be taken up by migratory cDC1 and cDC2 to be transported to the mediastinal LNs following lung infection (75, 76). Notably, our data showed that all the DCs could cross-present model antigen (SIINFEKL), slightly better by migratory cDC subsets, insinuating that the CD8^+^ T cell responses, in our model, are mainly governed by the uptake of antigen by the dominant subset of DC. Our study was in contrast with the others where cDC1 subset was instrumental subset for cross-presentation to CD8^+^ T cells. For example, the cutaneous melanoma antigens were cross-presented preferentially by migratory CD103^+^XCR1^+^ and CD103^-^XCR1^+^ skin-derived DC (77), and migratory cDC1 dominantly cross-presented herpes simplex virus and skin-derived self-antigens (47).

The activation of dendritic cells coupled with upregulation of costimulatory molecules is necessary for optimal priming of T cells, including CD8^+^ T cells. Our study showed that antigen uptake is DC subset-dependent, but cross-presentation was largely intact, suggesting that all DCs can cross-present in our model. The provision of cross-presentation ability improvement to increase the vaccine efficacy may not be contentious in our model, as the vaccination induces sterilizing immunity mediated by CD8^+^ T cells. Further, vaccination did not alter the expression of MHC-I and MHC-II molecules on DC subsets, possibly due to the nature of the cells, i.e., the constitutive expression of MHC molecules and optimal induction of T cell responses with that threshold level of expression. However, the expression of MHC molecules among different subsets differed: highest expression levels on migratory DCs, followed by resident DCs, and lower expression levels on moDC, and the MHC expression patterns on different DCs were in line with published studies (13). However, we did see a disparate pattern of CD80 and CD86 expression on DC subsets. Although both costimulatory molecules were significantly upregulated on many DC subsets, we found a significant fold increase of CD86 over CD80. Although CD80 and CD86 interact with CD28 and CTLA-4 ligands to regulate T cell responses, the studies have shown that CD86 has more potential in driving immune activation and T cell proliferation than CD80, which has a major role in immune tolerance (78–80). Interestingly, we did not find any differences in expression levels of CD40 on all DC subsets following vaccination.

CD4^+^ T cells are aptly called helper cells due to their myriad helper functions for immunity and tolerance. Apart from helping B cells secrete high-affinity antibodies and form long-lived memory and helping innate immune cells to enhance their direct effector functions, CD4^+^ T cells help in CD8^+^ cell activation, effector functions, and memory homeostasis (81, 82). Further, CD4^+^ T cells help CD8^+^ T cells by licensing dendritic cells, especially the cDC1 subset, by enhancing cross-presentation (19, 23, 24, 62, 83) and by secreting IL-2 (81, 84). The dendritic cell licensing further helps CD8^+^ T cell programming appropriately (85–87). However, we have undeniably shown that our model of fungal vaccination induces robust effector CD8^+^ T cells mediating sterilizing immunity following lethal challenge and programs to generate and maintain stable, long-lived memory independent of CD4^+^ T cells (32, 39–42). In line with this, we did not find upregulation of CD40, the “licensing molecule”, following the vaccination, nor was it affected by the presence of CD4^+^ T cells. Further, we found that CD4^+^ T cells were dispensable for DC responses, upregulation of costimulatory molecules, and antigen presentation. Similarly, CD4^+^ T cells did not influence the expression levels of MHC-I molecules in any of the DC subsets in our vaccine model. The enhanced number and ability of DC subsets may be due to the arsenal of immunostimulatory PAMPs, which directly activate the DCs and bypass the requirement for CD4^+^ T cell help (8, 9, 88–91).

## Conclusion

Our study showed that fungal vaccination significantly enhanced the frequencies, numbers, activation, fungal antigen uptake, and antigen cross-presenting ability of cDC2 subsets over cDC1 subsets. The CD4^+^ T cell-help was dispensable for the dendritic cell responses and retained the cDC2 dominant phenotype. Thus, our study offers new insights into type of DC responses to a fungal vaccine that elicits long-lasting CD8^+^ T-cell memory providing sterilizing immunity and help develop efficacious and safer vaccine platforms tailored for immunocompromised individuals.

## Data Availability

The original contributions presented in the study are included in the article/**Supplementary Material**. Further inquiries can be directed to the corresponding author/s.

## Supplementary material

Supplementary Figure 1 Dynamics of dendritic cell subsets, monocytes, and neutrophils following subcutaneous fungal vaccination. Naïve C57BL/6 mice after CD4 depletion were vaccinated subcutaneously with live attenuated strain (#55) of Blastomyces dermatitidis (∼2x10^5^ CFUs). The draining lymph nodes (dLN) were harvested on day 5 post-vaccination and stained for dendritic cell (DC) subsets to be analyzed by flow cytometry. The bar graphs depict the frequency (among lineage-ve cells) of resident cDCs, migratory cDCs, monocyte-derived DCs, langerin+ DCs, monocytes, and neutrophils. Data is representative of at least four independent experiments. N=5-8 mice/group. Values are in Mean ± SD. p*≤0.05, p**≤0.01, and p***≤0.001. Mice were injected with GK1.5 (200 µg/mouse) throughout the experiment to deplete CD4^+^ T cells.

Supplementary Figure 2 Kinetics of langerin+ DCs following subcutaneous fungal vaccination. Naïve C57BL/6 mice (CD4 depleted) were vaccinated s.c. with live attenuated strain (#55) of Blastomyces dermatitidis (~2x10^5^ CFUs). Frequency (among lineage^-ve^ cells) kinetics of Langerin+ DCs. Data are representative of at least four independent experiments. Values are Mean ± SD. p*≤0.05 and p****≤0.0001. Mice were injected with GK1.5 (200 µg/mouse) throughout the experiment to deplete CD4^+^ T cells.

Supplementary Figure 3 MHC class-I and -II expression levels on dendritic cell subsets following fungal vaccination. The bar graphs show the expression levels (mean fluorescent intensity-MFI) of **(A)** MHC class I (H-2K^b^) and **(B)** MHC class II (I-A/I-E) on the surface of resident cDCs, migratory cDCs, and monocyte-derived DCs at day 5 post-fungal vaccination in C57BL/6 mice depleted of CD4^+^ T cells. Data are representative of at least two independent experiments. N=3-5 mice/group. Values are in Mean± SD. p*≤0.05, p**≤0.01, and p***≤0.001.

Supplementary Figure 4 Frequency of PKH26^+^ cells among DC subsets. Naïve CD4- were subcutaneously vaccinated with PKH26^+^ labeled or unlabeled live attenuated strain (#55) of Blastomyces dermatitidis (∼6-8x10^6^ CFUs). The bar graphs show the frequency of PKH^+^ cells among lineage -ve cells **(A)** and among the DC subsets **(B)** in draining lymph nodes at day 5 post-vaccination. The grouped bar graphs depict the distribution of PKH26^+^ yeast among the DC subsets in dLN, shown in absolute numbers at day 5 post-vaccination. Values are in mean ± SD of at least two independent experiments. N=3-5 mice/group..

Supplementary Figure 5 Role of CD4^+^ T cells in the kinetics of monocytes, the activation status of moDCs, and the expression of MHC-I (H-2K^b^) among DC subsets. Naïve CD4^+^ and CD4 depleted C57BL/6 mice were vaccinated subcutaneously with live attenuated strain (#55) of Blastomyces dermatitidis (∼2x10^5^ CFUs). On indicated days post-vaccination, dLNs were harvested to analyze various DC subsets and monocytes by flow cytometry. The kinetics of **(A)** cell number and **(B)** frequencies (among lineage-ve cells) of monocytes are depicted. Values are Mean ± SD. N=3-5/mice/group/time-point. **(C)** The bar graphs show the activation status of monocyte-derived DCs marked by the expression of co-stimulatory molecules (CD80, CD86, and CD40) and surface marker MHC-II (I-A/I-E) and MHC-I at day 5 post-vaccination. **(D)** The bar graphs show the expression levels (MFI) of MHC class I (H-2Kb) on the surface of resident cDCs and migratory cDCs at day 5 post-fungal vaccination. Values are in Mean ± SD. N=3-5/mice/group. Data is representative of at least two independent experiments. p*≤0.05, p**≤0.01, and p***≤0.001, comparison between CD4^+^ and CD4^-^ vaccinated WT. Groups of mice were injected with GK1.5 (200 µg/mice) throughout the experiment to deplete CD4+ T cells..

Supplementary Figure 6 CD4^+^ T-cell depletion enhances cDC2 responses. Naïve C57BL/6 mice were administered with GK1.5 MAb or Isotype control (200 µg/mouse @3 days) were vaccinated subcutaneously with live attenuated strain (#55) of Blastomyces dermatitidis (∼2x10^5^ CFUs). On day 5 post-vaccination, dLNs were harvested to analyze various DC subsets by flow cytometry. The analysis was done as mentioned in **Figure 5** and **Supplementary Figure 5**. Values are Mean ± SD. N=5/mice/group. Percent, numbers, and MFI values are in Mean ± SD. p*≤0.05, p**≤0.01, and p****≤0.0001.

## References

[1] Bongomin F Gago S Oladele RO Denning DW. Global and multi-national prevalence of fungal diseases-estimate precision. J Fungi (Basel). (2017) 3(4):57. doi: 10.3390/jof3040057 29371573 PMC5753159

[2] Benedict K Whitham HK Jackson BR. Economic burden of fungal diseases in the United States. Open Forum Infect Dis. (2022) 9:ofac097. doi: 10.1093/ofid/ofac097 35350173 PMC8946773

[3] Denning DW. Global incidence and mortality of severe fungal disease. Lancet Infect Dis. (2024) 24:e428–38. doi: 10.1016/S1473-3099(23)00692-8 38224705

[4] Robbins N Wright GD Cowen LE. Antifungal drugs: the current armamentarium and development of new agents. Microbiol Spectr. (2016) 4(5). doi: 10.1128/microbiolspec.FUNK-0002-2016 27763259

[5] Srinivasan A Lopez-Ribot JL Ramasubramanian AK. Overcoming antifungal resistance. Drug Discovery Today Technol. (2014) 11:65–71. doi: 10.1016/j.ddtec.2014.02.005 PMC4031462 24847655

[6] Thomas CM Shae W Koestler D DeFor T Bahr NC Alpern JD. Antifungal drug price increases in the United States, 2000-2019. Mycoses. (2022) 65:859–65. doi: 10.1111/myc.13486 PMC9378588 35722703

[7] Peyclit L Yousfi H Rolain JM Bittar F. Drug repurposing in medical mycology: identification of compounds as potential antifungals to overcome the emergence of multidrug-resistant fungi. Pharm (Basel). (2021) 14(5):488. doi: 10.3390/ph14050488 PMC8161392 34065420

[8] Roy RM Klein BS. Dendritic cells in antifungal immunity and vaccine design. Cell Host Microbe. (2012) 11:436–46. doi: 10.1016/j.chom.2012.04.005 PMC3401965 22607797

[9] Kulkarni NA Nanjappa SG. Advances in dendritic-cell-based vaccines against respiratory fungal infections. Vaccines. (2024) 12:981. doi: 10.3390/vaccines12090981 39340013 PMC11435842

[10] Steinman RM. Dendritic cells and the control of immunity: enhancing the efficiency of antigen presentation. Mt Sinai J Med. (2001) 68:160–6. 11373688

[11] Eisenbarth SC. Dendritic cell subsets in T cell programming: location dictates function. Nat Rev Immunol. (2019) 19:89–103. doi: 10.1038/s41577-018-0088-1 30464294 PMC7755085

[12] Cabeza-Cabrerizo M Cardoso A Minutti CM Pereira da Costa M Reis e Sousa C. Dendritic cells revisited. Annu Rev Immunol. (2021) 39:131–66. doi: 10.1146/annurev-immunol-061020-053707 33481643

[13] Merad M Sathe P Helft J Miller J Mortha A. The dendritic cell lineage: ontogeny and function of dendritic cells and their subsets in the steady state and the inflamed setting. Annu Rev Immunol. (2013) 31:563–604. doi: 10.1146/annurev-immunol-020711-074950 23516985 PMC3853342

[14] Caro AA Deschoemaeker S Allonsius L Coosemans A Laoui D. Dendritic cell vaccines: A promising approach in the fight against ovarian cancer. Cancers (Basel). (2022) 14(16):4037. doi: 10.3390/cancers14164037 36011029 PMC9406463

[15] Mueller SN. Spreading the load: Antigen transfer between migratory and lymph node-resident dendritic cells promotes T-cell priming. Eur J Immunol. (2017) 47:1798–801. doi: 10.1002/eji.201747248 28845904

[16] Ersland K Wuthrich M Klein BS. Dynamic interplay among monocyte-derived, dermal, and resident lymph node dendritic cells during the generation of vaccine immunity to fungi. Cell Host Microbe. (2010) 7:474–87. doi: 10.1016/j.chom.2010.05.010 PMC2896205 20542251

[17] Backer RA Probst HC Clausen BE. Classical DC2 subsets and monocyte-derived DC: Delineating the developmental and functional relationship. Eur J Immunol. (2023) 53:e2149548. doi: 10.1002/eji.202149548 36642930

[18] Gardner A Ruffell B. moDCs, less problems. Immunity. (2018) 48:6–8. doi: 10.1016/j.immuni.2017.12.017 29343441

[19] Bennett SRM Carbone FR Karamalis F Flavell RA Miller JF Heath WR. Help for cytotoxic-T-cell responses is mediated by CD40 signalling. Nature. (1998) 393:478–80. doi: 10.1038/30996 9624004

[20] Smith CM Wilson NS Waithman J Villadangos JA Carbone FR Heath WR. Cognate CD4(+) T cell licensing of dendritic cells in CD8(+) T cell immunity. Nat Immunol. (2004) 5:1143–8. doi: 10.1038/ni1129 15475958

[21] Laidlaw BJ Craft JE Kaech SM. The multifaceted role of CD4(+) T cells in CD8(+) T cell memory. Nat Rev Immunol. (2016) 16:102–11. doi: 10.1038/nri.2015.10 PMC4860014 26781939

[22] Thaiss CA Semmling V Franken L Wagner H Kurts C. Chemokines: a new dendritic cell signal for T cell activation. Front Immunol. (2011) 2:31. doi: 10.3389/fimmu.2011.00031 22566821 PMC3342358

[23] Schoenberger SP Toes RE van der Voort EI Offringa R. & Melief, C.J. T-cell help for cytotoxic T lymphocytes is mediated by CD40-CD40L interactions. Nature. (1998) 393:480–3. doi: 10.1038/31002 9624005

[24] Ferris ST Durai V Wu R Theisen DJ Ward JP Bern MD. cDC1 prime and are licensed by CD4(+) T cells to induce anti-tumour immunity. Nature. (2020) 584:624–9. doi: 10.1038/s41586-020-2611-3 PMC7469755 32788723

[25] Theisen D Murphy K. The role of cDC1s *in vivo*: CD8 T cell priming through cross-presentation. F1000Res. (2017) 6:98. doi: 10.12688/f1000research 28184299 PMC5288679

[26] Embgenbroich M Burgdorf S. Current concepts of antigen cross-presentation. Front Immunol. (2018) 9:1643. doi: 10.3389/fimmu.2018.01643 30061897 PMC6054923

[27] Flinsenberg TW Spel L Jansen M Koning D de Haar C Plantinga M. Cognate CD4 T-cell licensing of dendritic cells heralds anti-cytomegalovirus CD8 T-cell immunity after human allogeneic umbilical cord blood transplantation. J Virol. (2015) 89:1058–69. doi: 10.1128/JVI.01850-14 PMC4300625 25378489

[28] Nakanishi Y Lu B Gerard C Iwasaki A. CD8(+) T lymphocyte mobilization to virus-infected tissue requires CD4(+) T-cell help. Nature. (2009) 462:510–3. doi: 10.1038/nature08511 PMC2789415 19898495

[29] Wu R Ohara RA Jo S Liu TT Ferris ST Ou F. Mechanisms of CD40-dependent cDC1 licensing beyond costimulation. Nat Immunol. (2022) 23:1536–50. doi: 10.1038/s41590-022-01324-w PMC9896965 36271147

[30] Nanjappa SG Heninger E Wuthrich M Sullivan T Klein B. Protective antifungal memory CD8(+) T cells are maintained in the absence of CD4(+) T cell help and cognate antigen in mice. J Clin Invest. (2012) 122:987–99. doi: 10.1172/JCI58762 PMC3287218 22354169

[31] Wuthrich M Filutowicz HI Warner T Deepe GS Jr. Klein BS. Vaccine immunity to pathogenic fungi overcomes the requirement for CD4 help in exogenous antigen presentation to CD8+ T cells: implications for vaccine development in immune-deficient hosts. J Exp Med. (2003) 197:1405–16. doi: 10.1084/jem.20030109 PMC2193905 12782709

[32] Nanjappa SG Klein BS. Vaccine immunity against fungal infections. Curr Opin Immunol. (2014) 28:27–33. doi: 10.1016/j.coi.2014.01.014 24583636 PMC4037332

[33] Zheng M Ramsay AJ Robichaux MB Kliment C Crowe C Rapaka RR. CD4+ T cell-independent DNA vaccination against opportunistic infections. J Clin Invest. (2005) 115:3536–44. doi: 10.1172/JCI26306 PMC1288835 16308571

[34] Fierer J Waters C Walls L. Both CD4+ and CD8+ T cells can mediate vaccine-induced protection against Coccidioides immitis infection in mice. J Infect Dis. (2006) 193:1323–31. doi: 10.1086/jid.2006.193.issue-9 16586371

[35] Kolls JK. IFN-gamma and CD8+ T cells restore host defenses against Pneumocystis carinii in mice depleted of CD4+ T cells. J Immunol. (1999) 162:2890–4. doi: 10.4049/jimmunol.162.5.2890 10072538

[36] Lindell DM Moore TA McDonald RA Toews GB Huffnagle GB. Generation of antifungal effector CD8+ T cells in the absence of CD4+ T cells during Cryptococcus neoformans infection. J Immunol. (2005) 174:7920–8. doi: 10.4049/jimmunol.174.12.7920 15944298

[37] Chiarella AP Arruda C Pina A Costa TA Ferreira RC Calich VL. The relative importance of CD4+ and CD8+T cells in immunity to pulmonary paracoccidioidomycosis. Microbes Infect. (2007) 9:1078–88. doi: 10.1016/j.micinf.2007.04.016 17692551

[38] Finkel-Jimenez B Wuthrich M Klein BS. BAD1, an essential virulence factor of Blastomyces dermatitidis, suppresses host TNF-alpha production through TGF-beta-dependent and -independent mechanisms. J Immunol. (2002) 168:5746–55. doi: 10.4049/jimmunol.168.11.5746 12023375

[39] Mudalagiriyappa S Sharma J Vieson MD Nanjappa SG. GM-CSF(+) Tc17 cells are required to bolster vaccine immunity against lethal fungal pneumonia without causing overt pathology. Cell Rep. (2022) 41:111543. doi: 10.1016/j.celrep.2022.111543 36288707 PMC9641983

[40] Nanjappa SG Heninger E Wuthrich M Gasper DJ Klein BS. Tc17 cells mediate vaccine immunity against lethal fungal pneumonia in immune deficient hosts lacking CD4+ T cells. PloS Pathog. (2012) 8:e1002771. doi: 10.1371/journal.ppat.1002771 22829762 PMC3400565

[41] Nanjappa SG McDermott AJ Fites JS Galles K Wüthrich M Deepe GS Jr. Antifungal Tc17 cells are durable and stable, persisting as long-lasting vaccine memory without plasticity towards IFNgamma cells. PloS Pathog. (2017) 13:e1006356. doi: 10.1371/journal.ppat.1006356 28542595 PMC5456400

[42] Sharma J Mudalagiriyappa S Nanjappa SG. T cell responses to control fungal infection in an immunological memory lens. Front Immunol. (2022) 13:905867. doi: 10.3389/fimmu.2022.905867 36177012 PMC9513067

[43] Nanjappa SG Mudalagiriyappa S Fites JS Suresh M Klein BS. CBLB constrains inactivated vaccine-induced CD8(+) T cell responses and immunity against lethal fungal pneumonia. J Immunol. (2018) 201:1717–26. doi: 10.4049/jimmunol.1701241 PMC6125178 30054317

[44] Kedl RM Lindsay RS Finlon JM Lucas ED Friedman RS Tamburini BAJ. Migratory dendritic cells acquire and present lymphatic endothelial cell-archived antigens during lymph node contraction. Nat Commun. (2017) 8:2034. doi: 10.1038/s41467-017-02247-z 29229919 PMC5725486

[45] Amon L Dudziak D Backer RA Clausen BE Gmeiner C Heger L. Guidelines for DC preparation and flow cytometry analysis of mouse lymphohematopoietic tissues. Eur J Immunol. (2023) 53:e2249893. doi: 10.1002/eji.202249893 36563125

[46] Meiser P Knolle MA Hirschberger A de Almeida GP Bayerl F Lacher S. A distinct stimulatory cDC1 subpopulation amplifies CD8(+) T cell responses in tumors for protective anti-cancer immunity. Cancer Cell. (2023) 41:1498–1515 e1410. doi: 10.1016/j.ccell.2023.06.008 37451271

[47] Bedoui S Whitney PG Waithman J Eidsmo L Wakim L Caminschi I. Cross-presentation of viral and self antigens by skin-derived CD103+ dendritic cells. Nat Immunol. (2009) 10:488–95. doi: 10.1038/ni.1724 19349986

[48] Allan RS Waithman J Bedoui S Jones CM Villadangos JA Zhan Y. Migratory dendritic cells transfer antigen to a lymph node-resident dendritic cell population for efficient CTL priming. Immunity. (2006) 25:153–62. doi: 10.1016/j.immuni.2006.04.017 16860764

[49] Wuthrich M Gern B Hung CY Ersland K Rocco N Pick-Jacobs J. Vaccine-induced protection against 3 systemic mycoses endemic to North America requires Th17 cells in mice. J Clin Invest. (2016) 126:795. doi: 10.1172/JCI85788 PMC4731166 26829626

[50] Leon B Lopez-Bravo M Ardavin C. Monocyte-derived dendritic cells formed at the infection site control the induction of protective T helper 1 responses against Leishmania. Immunity. (2007) 26:519–31. doi: 10.1016/j.immuni.2007.01.017 17412618

[51] Crozat K Tamoutounour S Vu Manh TP Fossum E Luche H Ardouin L. Cutting edge: expression of XCR1 defines mouse lymphoid-tissue resident and migratory dendritic cells of the CD8alpha+ type. J Immunol. (2011) 187:4411–5. doi: 10.4049/jimmunol.1101717 21948982

[52] Salomon B Cohen JL Masurier C Klatzmann D. Three populations of mouse lymph node dendritic cells with different origins and dynamics. J Immunol. (1998) 160:708–17. doi: 10.4049/jimmunol.160.2.708 9551906

[53] Tatsumi N Kumamoto Y. Role of mouse dendritic cell subsets in priming naive CD4 T cells. Curr Opin Immunol. (2023) 83:102352. doi: 10.1016/j.coi.2023.102352 37276821 PMC10524374

[54] Leon B Ardavin C. Monocyte-derived dendritic cells in innate and adaptive immunity. Immunol Cell Biol. (2008) 86:320–4. doi: 10.1038/icb.2008.14 18362945

[55] Nakano H Lin KL Yanagita M Charbonneau C Cook DN Kakiuchi T. Blood-derived inflammatory dendritic cells in lymph nodes stimulate acute T helper type 1 immune responses. Nat Immunol. (2009) 10:394–402. doi: 10.1038/ni.1707 19252492 PMC2668134

[56] Kitano M Yamazaki C Takumi A Ikeno T Hemmi H Takahashi N. Imaging of the cross-presenting dendritic cell subsets in the skin-draining lymph node. Proc Natl Acad Sci U.S.A. (2016) 113:1044–9. doi: 10.1073/pnas.1513607113 PMC4743831 26755602

[57] Becker M Güttler S Bachem A Hartung E Mora A Jäkel A. Ontogenic, phenotypic, and functional characterization of XCR1(+) dendritic cells leads to a consistent classification of intestinal dendritic cells based on the expression of XCR1 and SIRPalpha. Front Immunol. (2014) 5:326. doi: 10.3389/fimmu.2014.00326 25120540 PMC4112810

[58] Dorner BG Dorner MB Zhou X Opitz C Mora A Güttler S. Selective expression of the chemokine receptor XCR1 on cross-presenting dendritic cells determines cooperation with CD8+ T cells. Immunity. (2009) 31:823–33. doi: 10.1016/j.immuni.2009.08.027 19913446

[59] Manolova V Flace A Bauer M Schwarz K Saudan P Bachmann MF. Nanoparticles target distinct dendritic cell populations according to their size. Eur J Immunol. (2008) 38:1404–13. doi: 10.1002/eji.200737984 18389478

[60] Kim MK Kim J. Properties of immature and mature dendritic cells: phenotype, morphology, phagocytosis, and migration. RSC Adv. (2019) 9:11230–8. doi: 10.1039/C9RA00818G PMC9063012 35520256

[61] Wilson NS El-Sukkari D Villadangos JA. Dendritic cells constitutively present self antigens in their immature state *in vivo* and regulate antigen presentation by controlling the rates of MHC class II synthesis and endocytosis. Blood. (2004) 103:2187–95. doi: 10.1182/blood-2003-08-2729 14604956

[62] Calzada-Fraile D Iborra S Ramírez-Huesca M Jorge I Dotta E Hernández-García E. Immune synapse formation promotes lipid peroxidation and MHC-I upregulation in licensed dendritic cells for efficient priming of CD8(+) T cells. Nat Commun. (2023) 14:6772. doi: 10.1038/s41467-023-42480-3 37880206 PMC10600134

[63] Lahoud MH Ahmet F Kitsoulis S Wan SS Vremec D Lee CN. Targeting antigen to mouse dendritic cells via Clec9A induces potent CD4 T cell responses biased toward a follicular helper phenotype. J Immunol. (2011) 187:842–50. doi: 10.4049/jimmunol.1101176 21677141

[64] Valencia-Hernandez AM Zillinger T Ge Z Tan PS Cozijnsen A I McFadden G. Complexing CpG adjuvants with cationic liposomes enhances vaccine-induced formation of liver T(RM) cells. Vaccine. (2023) 41:1094–107. doi: 10.1016/j.vaccine.2022.12.047 36609029

[65] Soto JA Gálvez NMS Andrade CA Pacheco GA Bohmwald K Berrios RV. The role of dendritic cells during infections caused by highly prevalent viruses. Front Immunol. (2020) 11:1513. doi: 10.3389/fimmu.2020.01513 32765522 PMC7378533

[66] Ng SL Teo YJ Setiagani YA Karjalainen K Ruedl C. Type 1 conventional CD103(+) dendritic cells control effector CD8(+) T cell migration, survival, and memory responses during influenza infection. Front Immunol. (2018) 9:3043. doi: 10.3389/fimmu.2018.03043 30622538 PMC6308161

[67] Moon HG Eccles JD Kim SJ Kim KH Kim YM Rehman J. Complement C1q essential for aeroallergen sensitization via CSF1R(+) conventional dendritic cells type 2. J Allergy Clin Immunol. (2023) 152:1141–1152 e1142. doi: 10.1016/j.jaci.2023.07.016 37562753 PMC10923196

[68] Schlitzer A McGovern N Teo P Zelante T Atarashi K Low D. IRF4 transcription factor-dependent CD11b+ dendritic cells in human and mouse control mucosal IL-17 cytokine responses. Immunity. (2013) 38:970–83. doi: 10.1016/j.immuni.2013.04.011 PMC3666057 23706669

[69] Kashem SW Igyarto BZ Gerami-Nejad M Kumamoto Y Mohammed JA Jarrett E. Candida albicans morphology and dendritic cell subsets determine T helper cell differentiation. Immunity. (2015) 42:356–66. doi: 10.1016/j.immuni.2015.01.008 PMC4343045 25680275

[70] Xu J Hissong R Bareis R Creech A Goughenour KD Freeman CM. Batf3-dependent orchestration of the robust Th1 responses and fungal control during cryptococcal infection, the role of cDC1. mBio. (2024) 15:e0285323. doi: 10.1128/mbio.02853-23 38349130 PMC10936214

[71] Van Prooyen N Henderson CA Hocking Murray D Sil A. CD103+ Conventional Dendritic Cells Are Critical for TLR7/9-Dependent Host Defense against Histoplasma capsulatum, an Endemic Fungal Pathogen of Humans. PloS Pathog. (2016) 12:e1005749. doi: 10.1371/journal.ppat.1005749 27459510 PMC4961300

[72] Balic A Smith KA Harcus Y Maizels RM. Dynamics of CD11c(+) dendritic cell subsets in lymph nodes draining the site of intestinal nematode infection. Immunol Lett. (2009) 127:68–75. doi: 10.1016/j.imlet.2009.09.001 19766674 PMC2789245

[73] Gerner MY Casey KA Kastenmuller W Germain RN. Dendritic cell and antigen dispersal landscapes regulate T cell immunity. J Exp Med. (2017) 214:3105–22. doi: 10.1084/jem.20170335 PMC5626399 28847868

[74] Tomura M Hata A Matsuoka S Shand FH Nakanishi Y Ikebuchi R. Tracking and quantification of dendritic cell migration and antigen trafficking between the skin and lymph nodes. Sci Rep. (2014) 4:6030. doi: 10.1038/srep06030 25112380 PMC4129424

[75] Krishnaswamy JK Gowthaman U Zhang B Mattsson J Szeponik L Liu D. Migratory CD11b(+) conventional dendritic cells induce T follicular helper cell-dependent antibody responses. Sci Immunol. (2017) 2(18):eaam9169. doi: 10.1126/sciimmunol.aam9169 29196450 PMC7847246

[76] Plantinga M Guilliams M Vanheerswynghels M Deswarte K Branco-Madeira F Toussaint W. Conventional and monocyte-derived CD11b(+) dendritic cells initiate and maintain T helper 2 cell-mediated immunity to house dust mite allergen. Immunity. (2013) 38:322–35. doi: 10.1016/j.immuni.2012.10.016 23352232

[77] Wylie B Seppanen E Xiao K Zemek R Zanker D Prato S. Cross-presentation of cutaneous melanoma antigen by migratory XCR1(+)CD103(-) and XCR1(+)CD103(+) dendritic cells. Oncoimmunology. (2015) 4:e1019198. doi: 10.1080/2162402X.2015.1019198 26405572 PMC4570138

[78] Fallarino F Fields PE Gajewski TF. B7-1 engagement of cytotoxic T lymphocyte antigen 4 inhibits T cell activation in the absence of CD28. J Exp Med. (1998) 188:205–10. doi: 10.1084/jem.188.1.205 PMC2525552 9653097

[79] Kennedy A Waters E Rowshanravan B Hinze C Williams C Janman D. Differences in CD80 and CD86 transendocytosis reveal CD86 as a key target for CTLA-4 immune regulation. Nat Immunol. (2022) 23:1365–78. doi: 10.1038/s41590-022-01289-w PMC9477731 35999394

[80] Hathcock KS Laszlo G Pucillo C Linsley P Hodes RJ. Comparative analysis of B7-1 and B7-2 costimulatory ligands: expression and function. J Exp Med. (1994) 180:631–40. doi: 10.1084/jem.180.2.631 PMC2191623 7519245

[81] Castellino F Germain RN. Cooperation between CD4+ and CD8+ T cells: when, where, and how. Annu Rev Immunol. (2006) 24:519–40. doi: 10.1146/annurev.immunol.23.021704.115825 16551258

[82] Biram A Shulman Z. T cell help to B cells: Cognate and atypical interactions in peripheral and intestinal lymphoid tissues. Immunol Rev. (2020) 296:36–47. doi: 10.1111/imr.v296.1 32557712

[83] Lei X de Groot DC Welters MJP de Wit T Schrama E van Eenennaam H. CD4(+) T cells produce IFN-I to license cDC1s for induction of cytotoxic T-cell activity in human tumors. Cell Mol Immunol. (2024) 21:374–92. doi: 10.1038/s41423-024-01133-1 PMC10978876 38383773

[84] Cousens LP Orange JS Biron CA. Endogenous IL-2 contributes to T cell expansion and IFN-gamma production during lymphocytic choriomeningitis virus infection. J Immunol. (1995) 155:5690–9. doi: 10.4049/jimmunol.155.12.5690 7499855

[85] Zammit DJ Cauley LS Pham QM Lefrancois L. Dendritic cells maximize the memory CD8 T cell response to infection. Immunity. (2005) 22:561–70. doi: 10.1016/j.immuni.2005.03.005 PMC2857562 15894274

[86] Palucka K Banchereau J Mellman I. Designing vaccines based on biology of human dendritic cell subsets. Immunity. (2010) 33:464–78. doi: 10.1016/j.immuni.2010.10.007 PMC2975953 21029958

[87] Querec TD Akondy RS Lee EK Cao W Nakaya HI Teuwen D. Systems biology approach predicts immunogenicity of the yellow fever vaccine in humans. Nat Immunol. (2009) 10:116–25. doi: 10.1038/ni.1688 PMC4049462 19029902

[88] Greyer M Whitney PG Stock AT Davey GM Tebartz C Bachem A. T cell help amplifies innate signals in CD8(+) DCs for optimal CD8(+) T cell priming. Cell Rep. (2016) 14:586–97. doi: 10.1016/j.celrep.2015.12.058 26774484

[89] Shedlock DJ Whitmire JK Tan J MacDonald AS Ahmed R Shen H. Role of CD4 T cell help and costimulation in CD8 T cell responses during Listeria monocytogenes infection. J Immunol. (2003) 170:2053–63. doi: 10.4049/jimmunol.170.4.2053 12574376

[90] Gow NAR Latge JP Munro CA. The fungal cell wall: structure, biosynthesis, and function. Microbiol Spectr. (2017) 5(3). doi: 10.1128/microbiolspec.FUNK-0035-2016 PMC11687499 28513415

[91] Hatinguais R Willment JA Brown GD. PAMPs of the fungal cell wall and mammalian PRRs. Curr Top Microbiol Immunol. (2020) 425:187–223. doi: 10.1007/82_2020_201 32180018

