# Supplementary figures and images for: Fungal immunization potentiates CD4+ T cell-independent cDC2 responses for cross-presentation
Source: Front Immunol. 2025 May 26;16:1602174. doi: 10.3389/fimmu.2025.1602174 (PMC12146322; doi:10.3389/fimmu.2025.1602174)

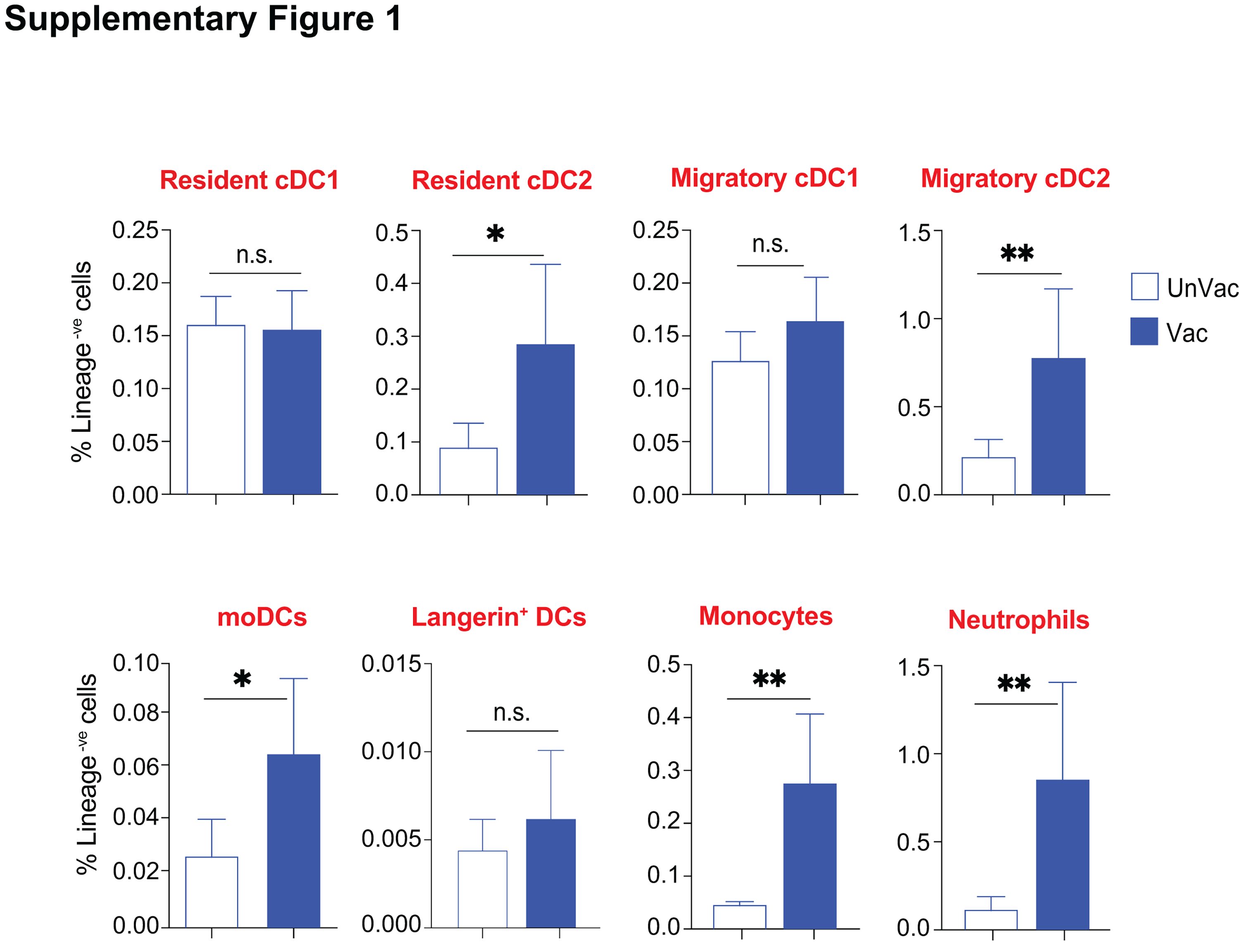

Supplement: Supplementary Figure 1 — Dynamics of dendritic cell subsets, monocytes, and neutrophils following subcutaneous fungal vaccination. Naïve C57BL/6 mice after CD4 depletion were vaccinated subcutaneously with live attenuated strain (#55) of Blastomyces dermatitidis (∼2x10^5^ CFUs). The draining lymph nodes (dLN) were harvested on day 5 post-vaccination and stained for dendritic cell (DC) subsets to be analyzed by flow cytometry. The bar graphs depict the frequency (among lineage-ve cells) of resident cDCs, migratory cDCs, monocyte-derived DCs, langerin+ DCs, monocytes, and neutrophils. Data is representative of at least four independent experiments. N=5-8 mice/group. Values are in Mean ± SD. p*≤0.05, p**≤0.01, and p***≤0.001. Mice were injected with GK1.5 (200 µg/mouse) throughout the experiment to deplete CD4+ T cells. [file Image1.tif]

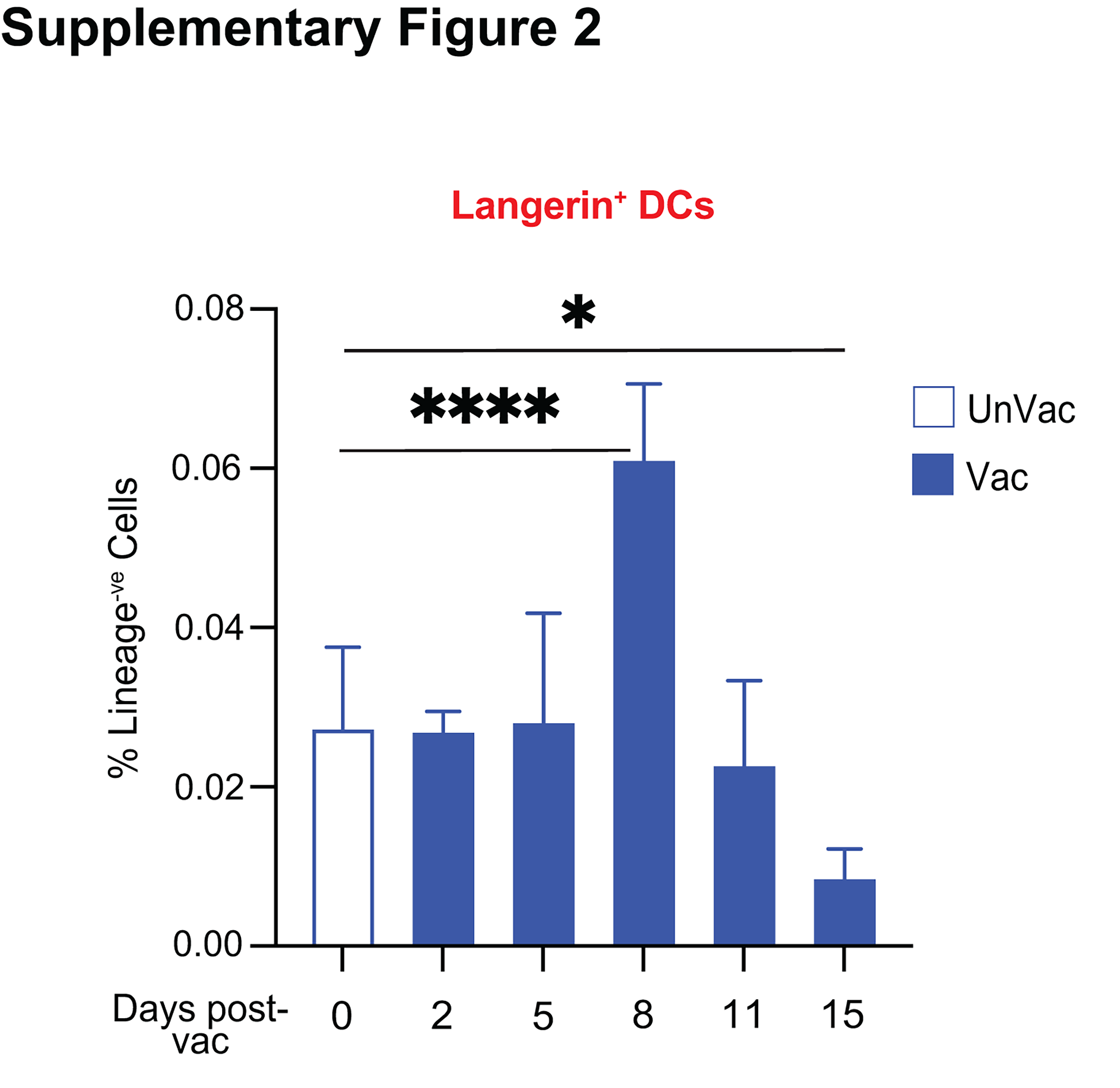

Supplement: Supplementary Figure 2 — Kinetics of langerin+ DCs following subcutaneous fungal vaccination. Naïve C57BL/6 mice (CD4 depleted) were vaccinated s.c. with live attenuated strain (#55) of Blastomyces dermatitidis (~2x10^5^ CFUs). Frequency (among lineage-ve cells) kinetics of Langerin+ DCs. Data are representative of at least four independent experiments. Values are Mean ± SD. p*≤0.05 and p****≤0.0001. Mice were injected with GK1.5 (200 µg/mouse) throughout the experiment to deplete CD4+ T cells. [file Image2.tif]

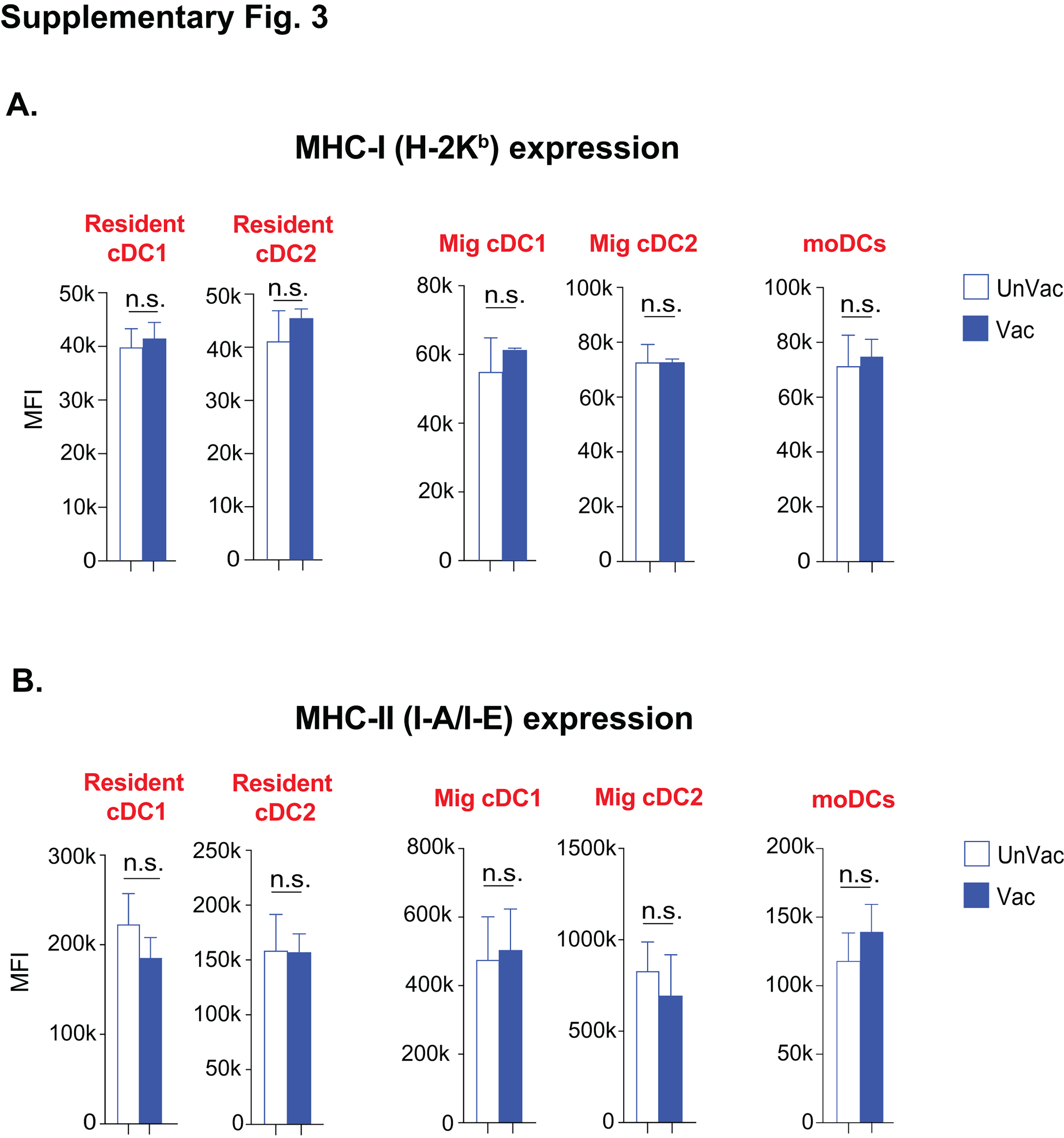

Supplement: Supplementary Figure 3 — MHC class-I and -II expression levels on dendritic cell subsets following fungal vaccination. The bar graphs show the expression levels (mean fluorescent intensity-MFI) of (A) MHC class I (H-2Kb) and (B) MHC class II (I-A/I-E) on the surface of resident cDCs, migratory cDCs, and monocyte-derived DCs at day 5 post-fungal vaccination in C57BL/6 mice depleted of CD4+ T cells. Data are representative of at least two independent experiments. N=3-5 mice/group. Values are in Mean± SD. p*≤0.05, p**≤0.01, and p***≤0.001. [file Image3.tif]

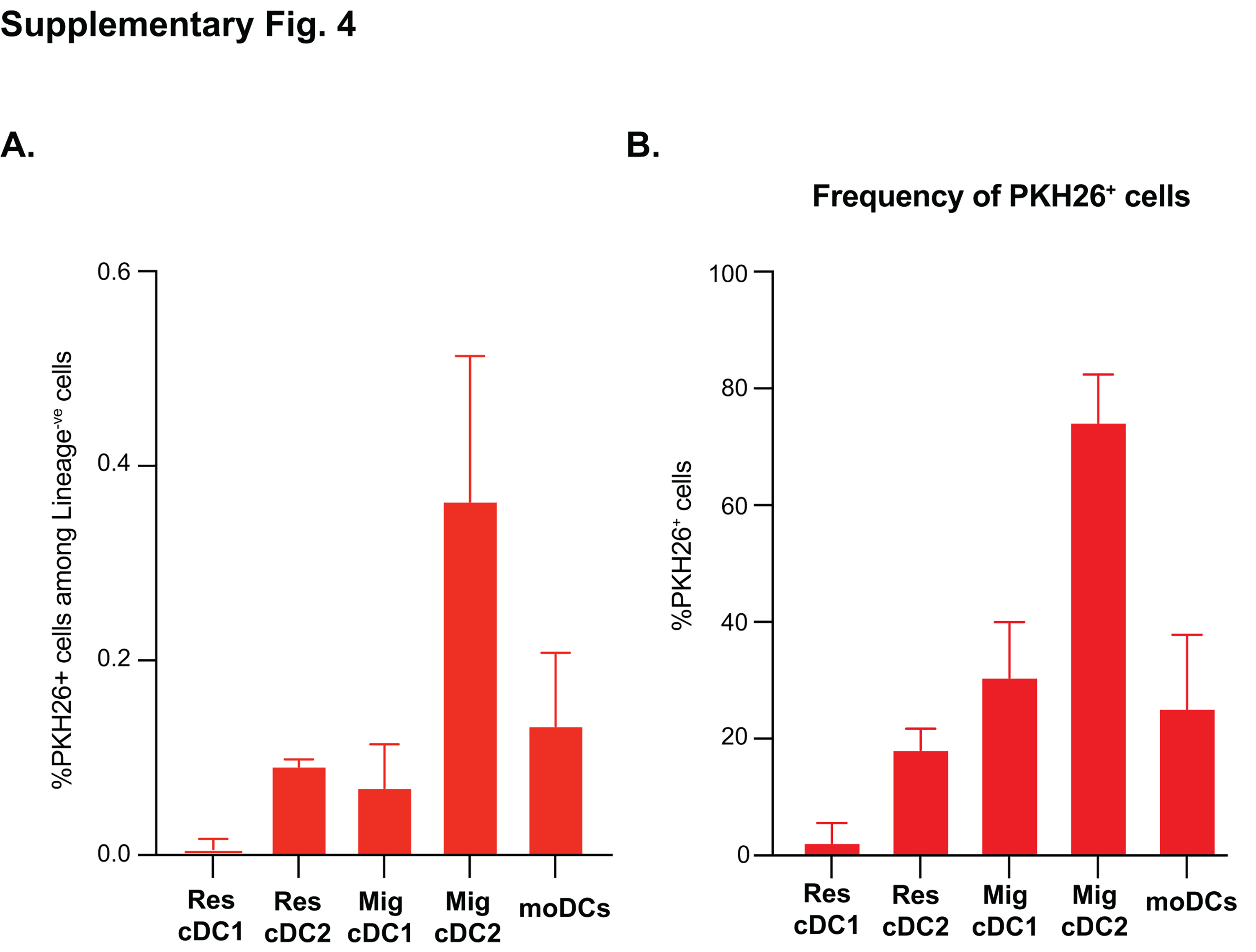

Supplement: Supplementary Figure 4 — Frequency of PKH26+ cells among DC subsets. Naïve CD4- were subcutaneously vaccinated with PKH26+ labeled or unlabeled live attenuated strain (#55) of Blastomyces dermatitidis (∼6-8x10^6^ CFUs). The bar graphs show the frequency of PKH+ cells among lineage -ve cells (A) and among the DC subsets (B) in draining lymph nodes at day 5 post-vaccination. The grouped bar graphs depict the distribution of PKH26+ yeast among the DC subsets in dLN, shown in absolute numbers at day 5 post-vaccination. Values are in mean ± SD of at least two independent experiments. N=3-5 mice/group.. [file Image4.tif]

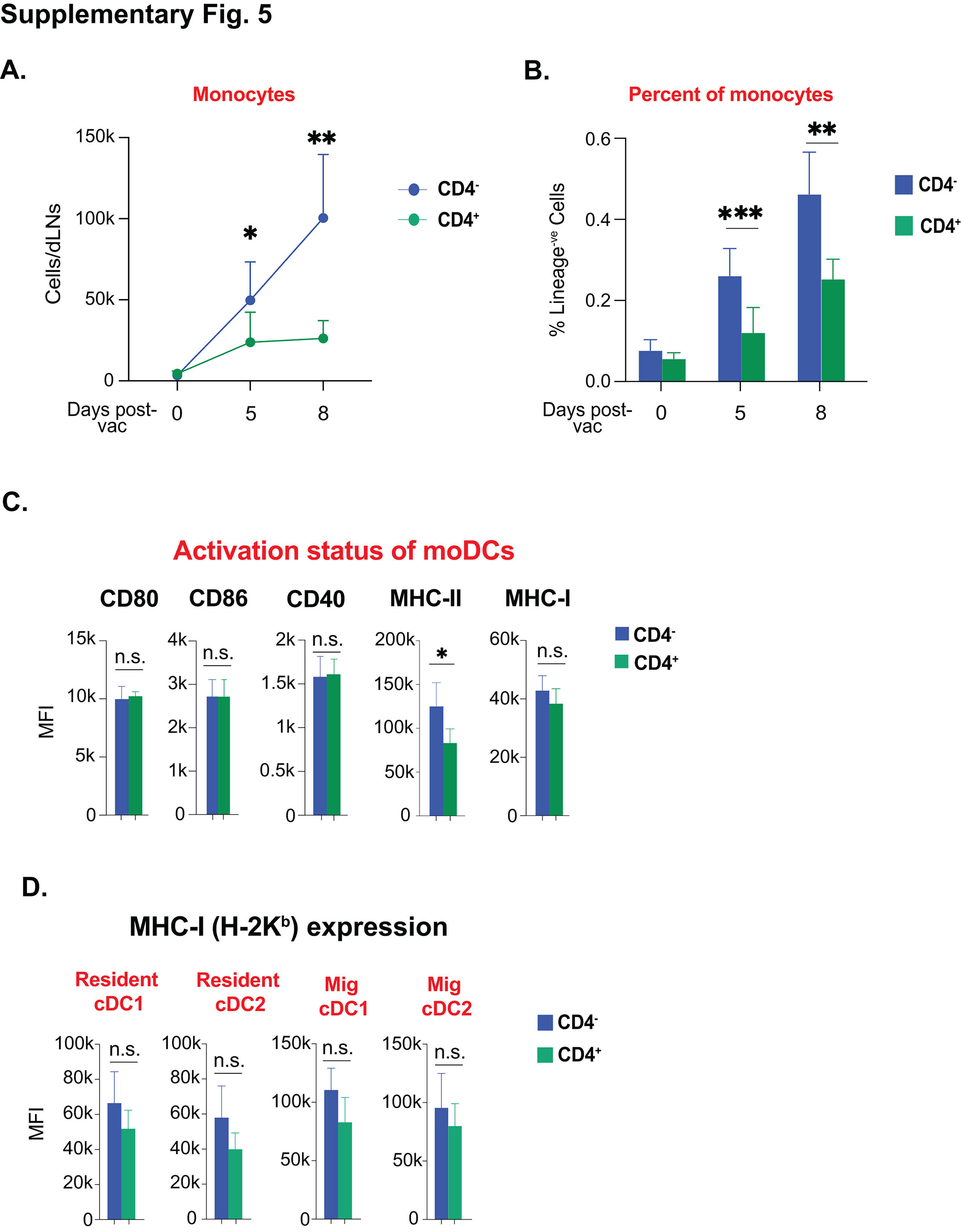

Supplement: Supplementary Figure 5 — Role of CD4+ T cells in the kinetics of monocytes, the activation status of moDCs, and the expression of MHC-I (H-2Kb) among DC subsets. Naïve CD4+ and CD4 depleted C57BL/6 mice were vaccinated subcutaneously with live attenuated strain (#55) of Blastomyces dermatitidis (∼2x10^5^ CFUs). On indicated days post-vaccination, dLNs were harvested to analyze various DC subsets and monocytes by flow cytometry. The kinetics of (A) cell number and (B) frequencies (among lineage-ve cells) of monocytes are depicted. Values are Mean ± SD. N=3-5/mice/group/time-point. (C) The bar graphs show the activation status of monocyte-derived DCs marked by the expression of co-stimulatory molecules (CD80, CD86, and CD40) and surface marker MHC-II (I-A/I-E) and MHC-I at day 5 post-vaccination. (D) The bar graphs show the expression levels (MFI) of MHC class I (H-2Kb) on the surface of resident cDCs and migratory cDCs at day 5 post-fungal vaccination. Values are in Mean ± SD. N=3-5/mice/group. Data is representative of at least two independent experiments. p*≤0.05, p**≤0.01, and p***≤0.001, comparison between CD4+ and CD4- vaccinated WT. Groups of mice were injected with GK1.5 (200 µg/mice) throughout the experiment to deplete CD4+ T cells.. [file Image5.tif]

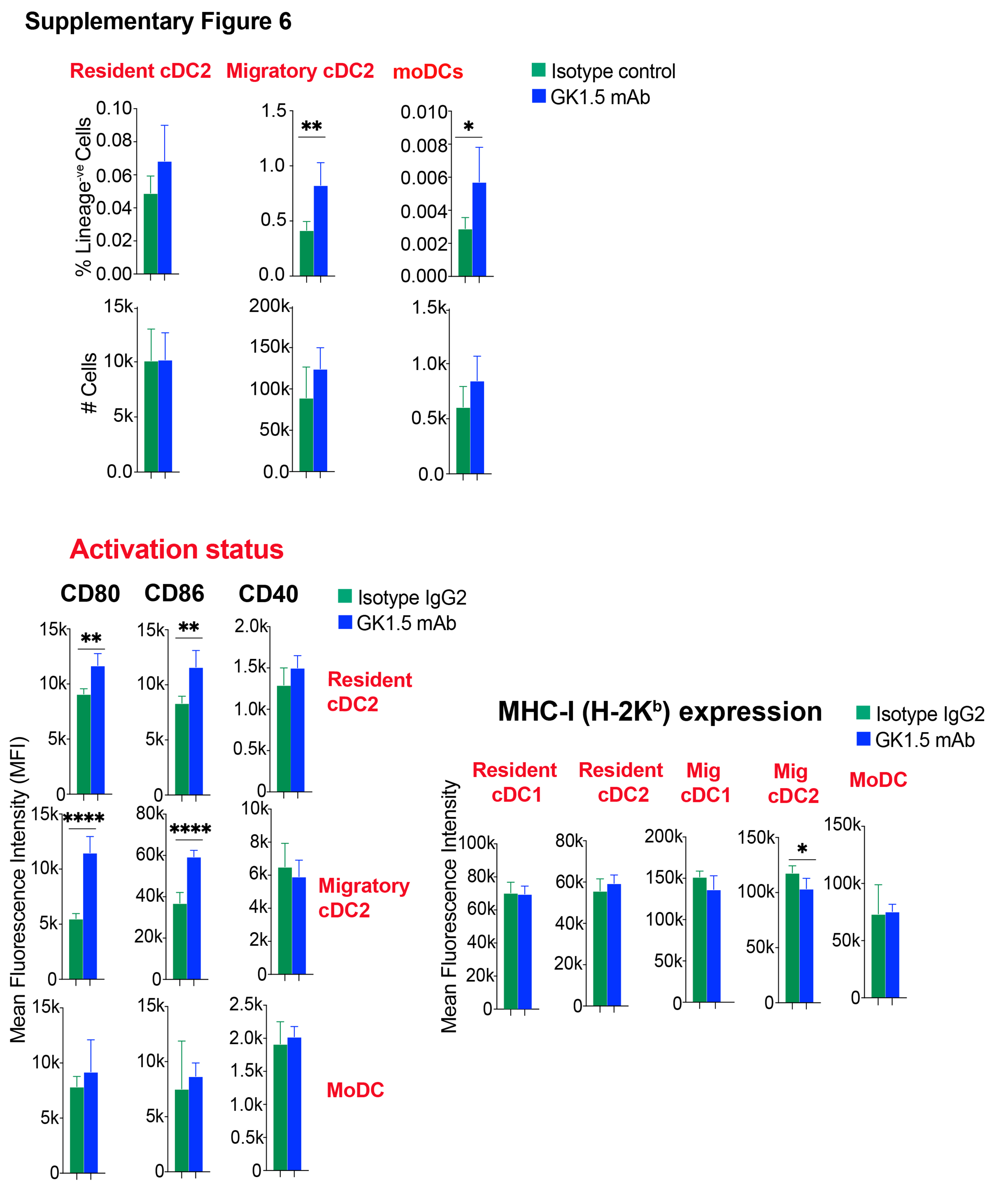

Supplement: Supplementary Figure 6 — CD4+ T-cell depletion enhances cDC2 responses. Naïve C57BL/6 mice were administered with GK1.5 MAb or Isotype control (200 µg/mouse @3 days) were vaccinated subcutaneously with live attenuated strain (#55) of Blastomyces dermatitidis (∼2x10^5^ CFUs). On day 5 post-vaccination, dLNs were harvested to analyze various DC subsets by flow cytometry. The analysis was done as mentioned in Figure 5 and Supplementary Figure 5 . Values are Mean ± SD. N=5/mice/group. Percent, numbers, and MFI values are in Mean ± SD. p*≤0.05, p**≤0.01, and p****≤0.0001. [file Image6.tif]
